# The Effect of Digital Mental Health Literacy Interventions on Mental Health: Systematic Review and Meta-Analysis

**DOI:** 10.2196/51268

**Published:** 2024-02-29

**Authors:** GeckHong Yeo, Stephanie M Reich, Nicole A Liaw, Elizabeth Yee Min Chia

**Affiliations:** 1 N.1 Institute for Health National University of Singapore Singapore Singapore; 2 School of Education University of California, Irvine Irvine, CA United States; 3 SHINE Children and Youth Services, Singapore Singapore Singapore

**Keywords:** review and meta-analysis, digital mental health literacy, digital mental health interventions, mental health functioning

## Abstract

**Background:**

Accelerated by technological advancements and the recent global pandemic, there is burgeoning interest in digital mental health literacy (DMHL) interventions that can positively affect mental health. However, existing work remains inconclusive regarding the effectiveness of DMHL interventions.

**Objective:**

This systematic review and meta-analysis investigated the components and modes of DMHL interventions, their moderating factors, and their long-term impacts on mental health literacy and mental health.

**Methods:**

We used a random-effects model to conduct meta-analyses and meta-regressions on moderating effects of DMHL interventions on mental health.

**Results:**

Using 144 interventions with 206 effect sizes, we found a moderate effect of DMHL interventions in enhancing distal mental health outcomes (standardized mean difference=0.42, 95% CI −0.10 to 0.73; *P*<.001) and a large effect in increasing proximal mental health literacy outcomes (standardized mean difference=0.65, 95% CI 0.59-0.74; *P*<.001). Uptake of DMHL interventions was comparable with that of control conditions, and uptake of DMHL interventions did not moderate the effects on both proximal mental health literacy outcomes and distal mental health outcomes. DMHL interventions were as effective as face-to-face interventions and did not differ by platform type or dosage. *DMHL plus* interventions (DMHL psychoeducation coupled with other active treatment) produced large effects in bolstering mental health, were more effective than *DMHL only* interventions (self-help DMHL psychoeducation), and were comparable with non-DMHL interventions (treatment as usual). DMHL interventions demonstrated positive effects on mental health that were sustained over follow-up assessments and were most effective in enhancing the mental health of emerging and older adults.

**Conclusions:**

For theory building, our review and meta-analysis found that DMHL interventions are as effective as face-to-face interventions. DMHL interventions confer optimal effects on mental health when DMHL psychoeducation is combined with informal, nonprofessional active treatment components such as skills training and peer support, which demonstrate comparable effectiveness with that of treatment as usual (client-professional interactions and therapies). These effects, which did not differ by platform type or dosage, were sustained over time. Additionally, most DMHL interventions are found in Western cultural contexts, especially in high-income countries (Global North) such as Australia, the United States, and the United Kingdom, and limited research is conducted in low-income countries in Asia and in South American and African countries. Most of the DMHL studies did not report information on the racial or ethnic makeup of the samples. Future work on DMHL interventions that target racial or ethnic minority groups, particularly the design, adoption, and evaluation of the effects of culturally adaptive DMHL interventions on uptake and mental health functioning, is needed. Such evidence can drive the adoption and implementation of DMHL interventions at scale, which represents a key foundation for practice-changing impact in the provision of mental health resources for individuals and the community.

**Trial Registration:**

PROSPERO International Prospective Register of Systematic Reviews CRD42023363995; https://www.crd.york.ac.uk/prospero/display_record.php?ID=CRD42023363995

## Introduction

### Background

Worldwide, mental illness is projected to have an economic cost of approximately US $6 trillion owing to poor productivity and negative health functioning [1]. The World Health Organization and World Federation for Mental Health have advocated for increasing global awareness of mental health [1]. To increase global understanding of and address mental health problems, researchers, policy makers, and mental health practitioners have long recognized the significance of individual- and society-level mental health literacy (MHL) [2]. MHL refers to “knowledge and beliefs about mental disorders which aid their recognition, management, or prevention” [3]. Low MHL in the general public is a key impediment to seeking mental health treatment [2]. A multifaceted construct, MHL comprises (1) understanding how to prevent mental illness, (2) understanding when a disorder is developing, (3) awareness of support and treatments for mental illness, (4) ability to effectively address mild mental health problems, and (5) mental health first aid skills to support others [4]. In more recent work, MHL has been expanded to include knowledge about (1) obtaining and maintaining good mental health, (2) understanding mental illnesses and treatments, (3) reducing mental illness–related stigma, (4) enhancing help-seeking efficacy or behaviors, and (5) enhancing help-seeking attitudes or intentions [5,6].

Unlike the extensive body of work on health literacy and its relationship to positive health outcomes [2,5,7], research on MHL is more recent and less established in how it relates to or affects mental health functioning [2]. Accelerated by technological advancements and the recent global pandemic, there is burgeoning interest in digital mental health interventions that can be delivered at scale and translate to real-world benefits [8,9]. In particular, recent work has highlighted the use of digital platforms (eg, web-based platforms, apps, and social media) as key facilitators for building digital MHL (DMHL), especially among young people [10,11]. Web-based pedagogies; diverse educational content; and timely interactions with peer supporters, trainers, mental health professionals, and people with common lived experiences of mental illness afforded by digital platforms can effectively promote DMHL [10,11]. As a nascent field, research on DMHL has primarily focused on and been incorporated into mental health interventions, with few observational or field studies documenting DMHL in naturalistic settings in relation to mental health functioning [12,13].

Existing work on DMHL interventions is scattered, and findings about the effects of DMHL interventions on mental health are mixed [14]. These inconsistent findings on DMHL interventions stem from how components of DMHL can vary across studies [15,16] and whether interventions assess the impact on proximal literacy outcomes only (facets of DMHL) [17,18], distal mental health outcomes only (mental health symptoms and conditions) [19], or both [15,16]. In particular, some interventions focus on DMHL as the primary intervening component, in which self-help psychoeducation is used to access information and learn about mental health [16,20]. Other interventions combine DMHL psychoeducation with types of treatment such as skills training; peer support; group discussions and activities; exercises such as diary entries and reflection logs; and informal, nonprofessional counselor interactions [15,21]. Digital mental health interventions that use DMHL as a primary or secondary component often include and are compared with other non-DMHL interventions. These non-DMHL interventions include treatment as usual with professional therapies (eg, cognitive behavioral therapy [CBT] and dialectical therapy) and skills training (eg, mindfulness [22,23]). Although receiving professional mental health treatment likely improves knowledge about mental health symptoms and management (eg, MHL), such interventions are not specifically focused on literacy. As such, they are categorized as non-DMHL interventions. Thus, it is unclear whether DMHL psychoeducation is sufficient or whether interventions need to incorporate DMHL with other active treatment components to improve individuals’ mental health.

Importantly, interventions with different DMHL components can have differential impacts on proximal and distal mental health outcomes [24,25]. DMHL interventions with DMHL psychoeducation can increase knowledge and beliefs about recognition, management, or prevention of mental disorders (the 5 facets of DMHL described previously) and likely have a greater impact on proximal (eg, knowledge, attitudes, and beliefs) than on distal (eg, improved mental health) [16] mental health outcomes. DMHL interventions coupled with active treatment components, on the other hand, may enhance both proximal and distal mental health outcomes [15]. Meta-analyses have focused on digital mental health treatment interventions such as professional therapies and skill-based training (eg, CBT and mindfulness meditation, respectively) [26]. There are no studies that evaluate the impact of DMHL interventions on both proximal and distal mental health outcomes or that consider psychoeducation alone or in conjunction with treatment. Research effort is required to synthesize findings across studies to draw inferences about the impact of DMHL interventions on mental health—specifically, whether DMHL interventions are fundamentally effective (ie, pretest-posttest DMHL intervention comparisons) and more effective than well-controlled conditions (ie, waitlist control and non-DMHL interventions).

### DMHL Versus Traditional Face-to-Face MHL Interventions

Scholars have argued that DMHL interventions combine ease of access and cost-efficiency with efficacy and are more effective on mental health [27,28]. DMHL interventions can overcome the shortcomings of traditional face-to-face MHL interventions, including low availability, a high threshold for participation, and substantial delivery costs [27,28]. DMHL interventions have several advantages [27,28]: (1) easy accessibility at any time and place; (2) assurance of anonymity to avoid stigmatization; (3) self-guidance for participants to work at their own pace and review materials as often as they want; (4) ability to reach individuals faster than traditional mental health services and prevent the onset of more severe mental health problems; and finally, (5) easy scalability, requiring only a small increase in resources to reach a greater proportion of the eligible population. Previous work has found that DMHL interventions combine ease of access and cost-efficiency with efficacy [27,28], whereas traditional face-to-face mental health treatment interventions that include DMHL components require close to 8 times more therapist time than digital ones [29].

DMHL interventions may also help populations that are not reached by existing traditional MHL approaches, particularly young people, whose lives are closely intertwined with digital media and are the primary users of web-based mental health resources [30]. DMHL interventions address 2 critical issues that traditional face-to-face MHL interventions encounter in targeting mental health, especially for youth mental health—concern for anonymity and limited reach [31]. For young people, the increased prevalence of mental health conditions, especially depression and anxiety [32,33], underscores the need to address major barriers to help-seeking behaviors, for instance, access, reach, and stigma [34]. Thus, DMHL interventions may play a particularly important role during adolescence and emerging adulthood as a cost-effective channel to intervene early in preventing mental illness and supporting mental health functioning [35].

An accumulating body of work indicates that DMHL interventions can reduce the public health burden by decreasing burnout among working professionals and distress among students [36,37]. Poor MHL is a primary factor that hinders the uptake of, engagement in, and adherence to mental health treatment prevention and intervention [2]. DMHL interventions have the potential to not only provide greater access and reach and reduce stigma but also improve MHL among the public [34]. Indeed, research has shown that improved MHL can address self-identifying mental health difficulties and enhance help-seeking intentions and behaviors, which are key to initial engagement in and subsequent adherence to treatment [2]. Thus, DMHL intervention is an upstream form of mental health prevention that can reduce the need for downstream intervention [1]. For instance, incorporating DMHL into mental health initiatives as a regular form of psychoeducation can target psychological readiness for enhancing mental health functioning [38]. DMHL may also function as a mental health resilience factor that protects individuals from and mediates the negative effects of adversity and risk factors for the development of psychopathology [39].

However, there is no conclusive evidence demonstrating the effectiveness of DMHL interventions compared with traditional face-to-face MHL interventions in bolstering mental health [40,41]. To establish evidence-based practices for DMHL interventions, an integrated and comprehensive investigation of DMHL features and affordances, as well as individual and contextual factors that amplify or attenuate the effectiveness of DMHL interventions on mental health, is needed [8,9]. The primary aim of our systematic review and meta-analysis was to evaluate the effectiveness of DMHL interventions on mental health by targeting several objectives. First, we addressed the lacunae in our current understanding of DMHL interventions by synthesizing findings across studies to ascertain how DMHL interventions compare with traditional face-to-face MHL interventions. Next, we assessed whether and how different DMHL components in DMHL interventions affect mental health outcomes. Third, we investigated the effectiveness of DMHL interventions on proximal MHL outcomes (eg, knowledge, attitudes, and beliefs) and distal mental health outcomes. Fourth, we established whether a strong inference can be drawn about the effectiveness of DMHL interventions—whether they are fundamentally effective (ie, pretest-posttest DMHL intervention comparisons) and more effective than well-controlled conditions. Finally, we amalgamated findings across studies to establish the long-term implications (carryover effects) of DMHL interventions for mental health functioning. A secondary aim was to identify moderating factors that amplify or attenuate the effectiveness of DMHL interventions, particularly DMHL features and affordances (new vs conventional platforms and dosages of intervention) and individual (sex, developmental differences, and severity of preexisting mental health conditions) and contextual (cultural contexts) factors.

### Effectiveness of DMHL on Uptake and Mental Well-Being

With the burgeoning interest in digital mental health interventions, which combine scalability, translation to real-world benefits, and cost-efficiency with efficacy [8,9], substantive research has been devoted to understanding whether and how digital modes of delivering mental health services, including DMHL, are superior to or comparable with traditional face-to-face delivery [8,9]. Efforts to synthesize studies to compare the mental health impact of DMHL and traditional modes of MHL interventions are warranted to provide conclusive evidence on the effectiveness of DMHL interventions. In particular, research has focused on the implementation and clinical effectiveness of digital mental health interventions broadly (but not on DMHL specifically) by assessing uptake (ie, engagement and adherence) and mental health outcomes [26], respectively. Although there is evidence that digital mental health interventions increase uptake and engagement and that greater intervention uptake enhances mental health functioning [26], similar evidence specific to DMHL is lacking. DMHL studies are scattered in their efforts to understand the impact of DMHL interventions in terms of both uptake and intervening in mental health outcomes and whether intervention uptake affects mental health outcomes.

Drawing from reviews and meta-analyses on digital mental health interventions (in the absence of similar work on DMHL), findings indicate that most interventions are implemented and evaluated in high-income countries (Global North) involving White samples [42-44]. Given existing mental health disparities that are compounded with the mental health costs of marginalization, racial and ethnic minority groups face greater mental health risks, including limited access to and greater barriers to engaging in digital treatment interventions for mental health [42-44]. Such findings underscore the need to attend to the ethnic or racial composition of the samples to understand the overall effectiveness of digital mental health interventions, including DMHL, on uptake and mental health outcomes. In considering impact, scholars and practitioners have called for attention to the long-term mental health implications of DMHL—specifically, the carryover effects of DMHL interventions on mental health functioning [36,45]. For instance, some DMHL interventions demonstrate no carryover effects [46,47], whereas others yield 3, 6, or 12 weeks of carryover effects [48,49]. Such evidence can drive the adoption and implementation of evidence-based DMHL interventions at scale, which represents a key foundation for practice-changing impacts in the provision of mental health resources for individuals and the community.

Our review of the DMHL intervention literature found that DMHL is typically the primary component—specifically, self-help DMHL psychoeducation that involves acquiring knowledge and information on mental health (hereafter referred to as *DMHL only* [16,20])—or a secondary component, incorporating DMHL psychoeducation with other active treatment components such as skills training; peer support; group discussions and activities; exercises such as diary entries and reflection logs; and informal, nonprofessional counselor interactions [15,21]. We refer to those interventions with DMHL as a secondary component as *DMHL plus*. To establish the effectiveness of DMHL interventions, it is necessary to consider the DMHL component of each intervention. DMHL interventions have predominantly focused on comparing mental health outcomes through the following DMHL components: (1) *DMHL only* (vs waitlist control [20]), (2) *DMHL plus* (vs waitlist control [15,16,50]), (3) *DMHL only* versus *DMHL plus* [21,51], and (4) *DMHL only* or *plus* versus non-DMHL (treatment as usual with professional therapies such as CBT and dialectical therapy [28,52]). Although receiving professional mental health treatment likely improves knowledge about mental health symptoms and management (eg, MHL), such interventions are not specifically focused on literacy. As such, they are categorized as non-DMHL interventions. Studies often compare the effectiveness of different intervention components on mental health outcomes with inconsistent findings. To ascertain whether DMHL interventions are fundamentally effective (pretest-posttest comparison) and whether stronger inferences about the effect of DMHL interventions can be drawn—that is, how they compare with control and other intervention conditions—we synthesized findings across three study designs: (1) pre- and postintervention comparison [53,54], (2) intervention group versus (waitlist) control group [49,54], and (3) DMHL intervention versus non-DMHL intervention [28,49].

Findings on the effectiveness of DMHL interventions on mental health outcomes are mixed, with some studies demonstrating positive effects [39,49] and others finding none [28,53]. A possible reason for these inconsistent findings is the contradictory ways of conceptualizing and operationalizing DMHL and mental health outcomes in DMHL interventions. Although most interventions include a DMHL component, few draw on the long-standing MHL paradigm [16,55]. Hence, for most interventions, it is often ambiguous which of the five facets of MHL were examined, including one or some combination of them [15,56]: (1) knowledge about obtaining and maintaining good mental health, (2) understanding mental illnesses and treatments, (3) reducing mental illness–related stigma, (4) enhancing help-seeking efficacy or behaviors, and (5) enhancing help-seeking attitudes or intentions [4].

Traditional face-to-face MHL interventions have found improvements in proximal mental health outcomes, particularly 1 or a combination of the 5 facets of MHL as well as distal mental health outcomes involving mental health conditions and functioning [56,57]. Extrapolating these findings to DMHL interventions, interventions targeting or incorporating different facets of MHL may have differential impacts on proximal and distal mental health outcomes [15,20]. However, there are insufficient DMHL studies that have investigated the 5 facets separately to distinguish their effects on mental health. Most studies have created composite variables of DMHL that comprise different combinations of the 5 facets [15,20]. Thus, with the available research on DMHL interventions, the synthesis of findings across studies is possible as a composite DMHL construct intervening in mental health to provide a conclusive understanding of the mental health impact of DMHL interventions.

Furthermore, DMHL interventions are inconsistent in whether they assess proximal outcomes only [17,18], distal outcomes only [19,54], or a combination of both increased literacy outcomes and better mental health conditions [21,28]. DMHL research often fails to acknowledge how changes in proximal DMHL outcomes factor in the effects of DMHL interventions on distal outcomes—mental health functioning [49,54]. Thus, research that distinguishes the effectiveness of DMHL interventions on both proximal DMHL outcomes and distal mental health outcomes is warranted. In addition, studies have obtained inconsistent findings on the effectiveness of DMHL interventions on the same mental health outcomes, such as depression [49,54], as well as across different mental health outcomes [58,59]. On the basis of the body of work on traditional face-to-face MHL interventions, researchers have argued for the importance of examining MHL with specific mental health outcomes (eg, eating disorders and depression) [2]. However, research on DMHL is in its infancy, and the existing literature contains insufficient estimates to test the impact of DMHL interventions on the range of mental health outcomes.

### Moderating Role of DMHL Features and Individual and Contextual Factors

To build a strong economic case for investing in digital mental health interventions that have clinical effectiveness (ie, reducing mental ill health and symptoms) broadly and on DMHL specifically, a key consideration is the dosage of the intervention [60]. Intervention durations that are too long or too short can affect its effectiveness [60], and typical digital mental health interventions that include DMHL components have a median dosage of 10 weeks [61,62]. However, it remains ambiguous whether this median of 10 weeks of intervention has a positive impact on mental health [60] and how it applies to the effectiveness of DMHL interventions. Hence, conclusive findings comparing different treatment dosages of DMHL interventions that demonstrate improvements or changes in mental health are necessary. Scholars have also argued that the features and affordances of digital platforms can factor into the effectiveness of digital mental health interventions by influencing users’ initial uptake and sustained engagement [8,9]. Accumulating research indicates that technological affordances can vary across platforms, from linear, static websites to more interactive social media platforms and mobile apps [8,9]. Specifically, DMHL interventions are commonly delivered through new and more conventional platforms. New platforms involving mobile apps, web-based or internet applications, and social media afford greater interactivity [20,54], whereas more conventional platforms, including films, videos, multimedia, and emails, afford lower or limited interactivity [17,18]. Thus, this review considered how the duration of the intervention and platform of delivery affect outcomes.

Drawing from meta-analyses and systematic reviews of digital mental health interventions, individual factors related to preexisting physical and mental health conditions can modulate the effectiveness of interventions [8]. Physical illnesses such as rheumatoid arthritis can interact and generate or exacerbate mental health problems and reduce the effectiveness of interventions [63]. The severity of one’s preexisting mental illness can limit intra- and interpersonal resources (ie, motivation and supportive relationships, respectively) for uptake of, engagement in, and adherence to digital mental health interventions, thus reducing their effectiveness [8]. Extending these arguments to DMHL interventions, some research has shown that individual factors, particularly being female and having a mental health problem, are associated with relatively high levels of MHL and reduced effects of DMHL interventions on mental health outcomes (a possible ceiling effect [64]). However, these moderating effects of sex and preexisting mental health conditions on the intervening effectiveness of DMHL interventions are tentative and not consistently documented across studies [64,65]. Thus, the characteristics of individuals need to be considered as important moderators.

Culture is a moderating contextual factor in the effectiveness of digital mental health interventions [66], but there is a dearth of research that investigates different cultures. Existing literature only allows for a comparison of the effects of DMHL interventions across Western and Eastern cultural contexts [67,68]. In more independence-oriented Western cultural contexts, values and norms are focused on developing a healthy sense of self, including positive mental health [67,68]. There is greater public awareness of mental health; less social stigma associated with help-seeking behaviors; and more concerted efforts to build mental health resiliency through the adoption and implementation of digital mental health interventions, including DMHL interventions [67,68]. Thus, in Western cultural contexts, greater MHL and a focus on mental health may amplify the effects of DMHL interventions. In contrast, most collectivistic, interdependence-oriented Asian societies are conservative in addressing mental health [67], and they promote MHL and positive mental health functioning in ways that differ from those of individualistic Western cultural contexts, for instance, cultivating interdependence relationships, relational harmony, and dialectical beliefs and emotions—a balanced state of opposites, including experiencing both positive and negative emotions, which are fused with and change into each other [68]. The increase in awareness of mental health issues is relatively recent, especially in contemporary Asian societies [69,70], which may be associated with weaker impacts of DMHL interventions on mental health [71]. The paucity of reviews and meta-analytic efforts that synthesize findings across international studies on digital mental health interventions makes findings about cultural context tentative and underscores the importance of this consideration in this study.

### Aims of This Review and Meta-Analysis

To our knowledge, no systematic review or meta-analysis has evaluated the effectiveness of DMHL interventions on mental well-being or the moderating factors between these 2. To this end, our efforts to synthesize findings across studies focused on the main mental health impacts of DMHL interventions. First, we ascertained how DMHL interventions compare with traditional face-to-face MHL interventions in affecting mental health outcomes. Second, we examined the effects of DMHL interventions on the implementation outcome of uptake and the proximal DMHL outcomes and distal mental health outcomes. In addition, we examined whether uptake of DMHL interventions affects the impact on the proximal DMHL outcomes and distal mental health outcomes. Third, we investigated the impact of DMHL interventions on mental health through the following DMHL components: (1) *DMHL only* (self-help DMHL psychoeducation that entails acquiring knowledge and information), (2) *DMHL plus* (DMHL psychoeducation combined with skills training; peer support; group discussions and activities; exercises such as diary entries and reflection logs; and informal, nonprofessional counselor interactions), (3) *DMHL only* versus non-DMHL (treatment as usual through professional therapies such as CBT and dialectical therapy), and (4) *DMHL plus* versus non-DMHL. Fourth, our review and meta-analytic efforts provide evidence for a stronger inference on the effectiveness of DMHL interventions on mental health functioning by combining and comparing results across three study designs: (1) pre- and postintervention comparison, (2) intervention group versus (waitlist) control group, and (3) DMHL intervention versus non-DMHL intervention. Fifth, we addressed the gap in the current literature regarding the long-lasting effectiveness of DMHL interventions on mental health by assessing carryover effects. Finally, we considered potential moderators of treatment effects, including dosage, platform, individual characteristics, and culture.

In addressing these main effects, our meta-analytic efforts conceptualized and operationalized DMHL as a composite construct to understand its mental health impacts as there are limited studies that have examined all 5 facets of DMHL and mental well-being conjointly and separately. Thus, there are insufficient studies (and number of effect sizes) to determine how the 5 facets of DMHL compare in their impacts on mental health. Although scholars have argued for the study of digital mental health interventions for specific mental health outcomes (eg, depression vs anxiety vs eating disorders [8,9]), the limited number of studies in the literature renders it impossible to study such specific effects, particularly for DMHL interventions. On the basis of the literature reviewed previously, we examined the following research questions (RQs) and hypotheses:

1. What is the effect of DMHL interventions on uptake as an implementation outcome? (RQ 1a)
2. What are the effects of DMHL interventions on proximal literacy outcomes (ie, DMHL outcomes) and distal mental health outcomes? (RQ 1b)
3. Does the uptake of DMHL interventions moderate the effects on proximal literacy outcomes and distal mental health outcomes? (RQ 1c)
4. How do DMHL interventions with different DMHL components or conditions compare in their impact on mental health (*DMHL only*, *DMHL plus*, *DMHL only* vs non-DMHL, and *DMHL plus* vs non-DMHL)? (RQ 2a)
5. Do DMHL interventions demonstrate a stronger inference on intervening in mental health functioning? Specifically, what are the effects of DMHL interventions in pretest-posttest comparisons, (waitlist) control groups versus intervention groups, and DMHL interventions versus non-DMHL interventions? (RQ 2b)
6. Are there sustained or carryover effects of DMHL interventions on mental health? (RQ 3)
7. DMHL interventions are as effective as traditional face-to-face MHL interventions in bolstering mental health (hypothesis 1).
8. DMHL interventions administered through new platforms that afford greater interactivity (ie, mobile apps, web-based or internet platforms, and social media) than more conventional platforms (ie, films, videos, multimedia, and emails) have greater positive impacts on mental health (hypothesis 2).
9. DMHL interventions with a dosage of 10 weeks are most effective in increasing mental health functioning (hypothesis 3).
10. DMHL interventions demonstrate reduced effects on mental health in female participants compared with male participants (hypothesis 4a).
11. DMHL interventions have a lower impact on mental health in individuals with greater severity of preexisting mental health conditions (hypothesis 4b).
12. DMHL interventions have greater effects in adolescents than in participants at other developmental stages (ie, emerging adulthood and older adulthood; hypothesis 4c).
13. The intervening effectiveness of DMHL interventions on mental health is greater in Western than in Eastern cultural contexts (hypothesis 5).

## Methods

This meta-analysis was conducted according to the guidelines from Quintana [72] and reported based on the latest version of the PRISMA (Preferred Reporting Items for Systematic Reviews and Meta-Analyses) guidelines [73]. A protocol was registered a priori following the PRISMA guidelines (PROSPERO registration CRD42023363995).

### Literature Search

With the assistance of a staff librarian at the first author’s affiliated institution, 2 research assistants independently used 3 search strategies to systematically identify studies on DMHL and mental health. The 3 search strategies; search terms that were developed using the Population, Intervention, Comparison, and Outcome search strategy; and full search strings are provided in Tables S1 to S3 in Multimedia Appendix 1.

### Study Selection

**Figure 1:**
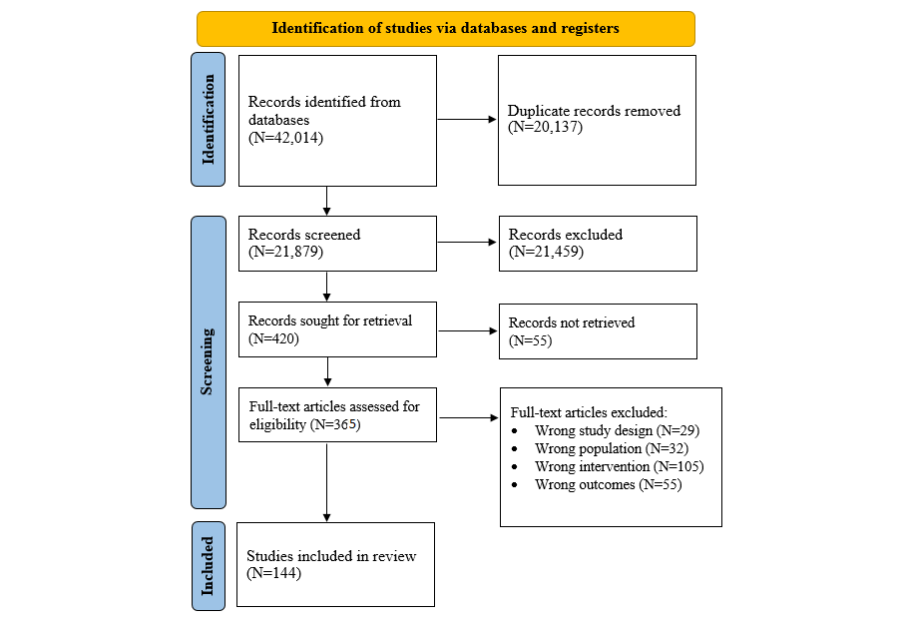
Flowchart of the study selection process.

Study screening was conducted using the Covidence software (Veritas Health Innovation) [74]. Figure 1 presents a flowchart of the study selection process. Of the 42,014 records identified in the initial searches, 20,137 (47.93%) were duplicates. First-stage screening of the 21,879 (52.08%) remaining records entailed checking of titles and abstracts by 2 reviewers, which led to the elimination of 21,459 (98.08%) of the 21,879 records. From the 420 (1.92%) of the 2,1879 records sought for retrieval, 55 (13.1%) of the 420 records were not retrieved due to the lack of an English full-text pdf article. The remaining 367 (87.4%) records selected for full text review were independently screened by another 2 reviewers with any discrepancies resolved through consensus. A third reviewer was contacted if consensus could not be reached. Inclusion and exclusion criteria were established a priori and are included in Tables S1 to S3 Multimedia Appendix 1.

A total of 146 studies with 208 effect sizes were included in our meta-analyses, with 2 (1.4%) field studies reporting relevant correlation coefficients and 144 (98.6%) intervention studies providing sufficient statistics to compute the standardized mean difference (SMD; Cohen *d*), which contributed 2 and 206 effect sizes, respectively. Although DMHL interventions are examined in field studies and as interventions in experimental studies, it is methodologically feasible to examine both field and experimental studies within the same meta-analysis [75,76]. However, we excluded the field studies (2/146, 1.4%) as they contributed only 2 effect size estimates, which are insufficient for a comparison of the relationship between DMHL and mental health across field and intervention studies.

### Data Extraction and Coding

In total, 2 independent reviewers (2 research assistants) extracted and coded data on multiple aspects (see Multimedia Appendix 2 [77-81] and Multimedia Appendix 3 [82,83] on study characteristics and summary and sample characteristics, respectively). The coders achieved 83% agreement on their codes. Any discrepancies in coding were discussed and resolved. Many of the interventions included in our meta-analysis (54/144, 37.5%) did not provide a measure of MHL. The 34% (49/144) of interventions that assessed DMHL outcomes primarily used the Mental Health Literacy Scale [84].

### Risk-of-Bias (Quality) Assessment

Studies included in the meta-analysis were independently evaluated for quality by 2 reviewers, with differences discussed and resolved. DMHL interventions were assessed using the instrument by Downs and Black [85] and the Cochrane Collaboration risk-of-bias tool [86]. We report these results in Multimedia Appendix 2.

### Multiple Dependent Effect Sizes and Computation of Effect Sizes

There were several instances in which the studies contributed multiple dependent effect sizes in our meta-analyses. Following the guidelines provided by Quintana [72], we dealt with this issue in 3 ways that we outlined in Figure S1 in Multimedia Appendix 4 [82,83].

### Publication Bias Analysis

A total of 3 analyses were used to ascertain publication bias, and the results are reported in Figure S1 in Multimedia Appendix 5.

### Data Analysis

Data were analyzed using RStudio (version 4.0.0; Post, PBC) with the *metafor* and *robumta* packages (version 3.02) [87-89].

## Results

### Overview

Summary and sample characteristics of the included DMHL intervention studies, as well as of traditional face-to-face MHL intervention studies, are presented in Tables S1 and S2 in Multimedia Appendix 2. We included a forest plot to visualize the effect sizes and CIs from the included studies with a computed summary effect size (Multimedia Appendix 5). In the following sections, we present the results that address each hypothesis and RQ.

### Effectiveness of DMHL Interventions in Enhancing Uptake and Mental Health

As hypothesized, DMHL interventions had similar effectiveness to that of traditional face-to-face MHL interventions in bolstering mental health (*Q_between_*=4.12; *P*=.18; hypothesis 1; Table S3 in Multimedia Appendix 2). Addressing RQ 1a, DMHL interventions versus control conditions had comparable effects on uptake (odds ratio 0.998, 95% CI 0.91-1.03; *P*<.001; Table S4 in Multimedia Appendix 2). For RQ 1b, we found a high effect of DMHL interventions in increasing proximal literacy outcomes (ie, 5 facets of DMHL), with a pooled effect size of SMD=0.65 (95% CI 0.59-0.74; *P*<.001), and a moderate effect in enhancing distal mental health outcomes, with a pooled effect size of SMD=0.42 (95% CI −0.10 to 0.73; *P*<.001). Unfortunately, few studies (2/144, 1.4%) differentiated the 5 facets of DMHL, and thus, we were unable to assess the effects of each DMHL facet on the outcomes. For RQ 1c*,* we found that uptake of DMHL interventions did not moderate the effect of the interventions on proximal literacy outcomes (*Q_between_*=1.07; *P*=.30). Subgroup comparisons of (1) DMHL interventions with baseline and completer samples that were similar in baseline and demographic characteristics, (2) DMHL interventions that did not provide information on baseline and completer samples’ baseline and demographic characteristics, and (3) DMHL interventions with baseline and completer samples that were different in baseline and demographic characteristics indicated no significant difference in impacts on MHL outcomes (*Q_between_*=1.19; *P*=.55). Similarly, our results revealed that the uptake of DMHL interventions did not moderate the effect of the interventions on distal mental health outcomes (*Q_between_*=0.17; *P*=.68). Subgroup comparisons of (1) DMHL interventions with baseline and completer samples that were similar in baseline and demographic characteristics, (2) DMHL interventions that did not provide information on baseline and completer samples’ baseline and demographic characteristics, and (3) DMHL interventions with baseline and completer samples that were different in baseline and demographic characteristics indicated no significant difference in impacts on mental health outcomes (*Q_between_*=5.21; *P*=.08).

For RQ 2a, we compared DMHL interventions with DMHL alone or with additional aspects (ie, *DMHL only* vs waitlist control conditions, *DMHL plus* vs waitlist control conditions, *DMHL only* vs *DMHL plus*, and *DMHL plus* vs non-DMHL). *Q* statistics analyses comparing these 4 conditions indicated that they differed significantly (*Q_between_*=9.45; *P*=.01). The effect size of *DMHL only* versus waitlist control was comparable with that of *DMHL plus* versus waitlist control but greater than that of *DMHL only* versus *DMHL plus* and *DMHL plus* versus non-DMHL (Table S1 in Multimedia Appendix 3). To address RQ 2b, we synthesized the effect sizes from 44.4% (64/144) of the DMHL interventions to elucidate whether we could draw stronger inferences about the effectiveness of DMHL interventions in increasing mental health. *Q* statistics analyses comparing the effect sizes of pretest-posttest DMHL intervention comparisons, waitlist control conditions versus DMHL interventions, and DMHL versus non-DMHL interventions revealed that they differed significantly (*Q_between_*=12.09; *P*<.001), with higher effect sizes for pretest-posttest DMHL intervention comparisons and waitlist control conditions versus DMHL interventions than for DMHL versus non-DMHL interventions. For RQ 3, subgroup comparison of DMHL interventions that assessed mental health outcomes at the postintervention time point and those with follow-up assessments indicated that they did not differ significantly (*Q_between_*=3.81; *P*=.06), with the effect sizes for interventions that assessed postintervention effects on mental health and those that assessed follow-up effects being comparable. Consistently, the carryover effects of DMHL interventions on mental health did not attenuate over time (*Q_between_*=1.65; *P*=.20; Table S1 in Multimedia Appendix 3); the positive effects of DMHL interventions on mental health remained notwithstanding longer follow-up assessments.

### Moderating Factors: DMHL Features, Individual Characteristics, and Cultural Contexts

We investigated potential moderators of DMHL interventions on mental health functioning (Table S1 in Multimedia Appendix 6 [90]) by performing subgroup analyses using meta-regressions to examine DMHL platform affordances (new interactive platforms, including mobile apps, web-based or internet platforms, and social media, vs conventional platforms, including films, videos, multimedia, and emails) and dosage of DMHL interventions as moderators. For dosage, we compared DMHL interventions of <10 weeks, 10 weeks, and >10 weeks. Contrary to hypothesis 2, DMHL interventions administered through new platforms that afford greater interactivity than more conventional platforms did not differ significantly in their impacts on mental health (*Q_between_*=2.51; *P*=.31). In contrast to hypothesis 3, we found that the dosage of the DMHL interventions did not moderate their effectiveness on mental health outcomes (*Q_between_*=2.13; *P*=.32), and similar positive intervening effects were found for all 3 dosage categories (Table S1 in Multimedia Appendix 3).

Developmental stages, including adolescence, emerging adulthood, and adulthood; sex, which was measured as the average proportion of participants in the sample who were female; and severity of mental health conditions at baseline were used as continuous predictors in testing their moderating effects. Contrary to hypothesis 4a, sex (*Q_between_*=2.01; *P*=.34) did not moderate the impact of DMHL interventions on mental health. Contrary to our hypothesis, DMHL interventions were more effective in enhancing the mental health of emerging and older adults than that of adolescents (hypothesis 4b; *Q_between_*=12.19; *P*=.001), and the severity of mental health conditions did not attenuate the effect of DMHL interventions on mental health (hypothesis 4c; *Q_between_*=0.29; *P*=.45; Table S1 in Multimedia Appendix 6).

The studies included in the meta-analyses involved 23 countries, but there were not enough studies from each country to allow for a comparison of effect sizes among individual countries. Instead, we followed the common approach of comparing Western and Eastern cultures [68,91]. Representatives of Western culture included Australia, Canada, Germany, the Netherlands, the United Kingdom, and the United States, whereas representatives of Eastern culture included China, Hong Kong, Pakistan, Singapore, and Taiwan. In contrast to hypothesis 5, we found that DMHL interventions conducted in Western and Eastern cultural contexts did not differ significantly in their effectiveness on mental health (*Q_between_*=1.13; *P*=.64; Table S1 in Multimedia Appendix 6).

## Discussion

### Principal Findings

The rise in mental health issues worldwide accelerated during the pandemic, highlighting the need for upstream mental health prevention [1]. A key approach to mental health prevention is to build individuals’ resiliency, which can protect them from and mediate the negative impacts of adversity and risk factors for the development of psychopathology [54]. Recent work on digital mental health interventions, particularly with DMHL, provides evidence for DMHL as a resilience factor that might mitigate stress and mental health conditions among working adults and students [36,37]. DMHL interventions combine ease of access with low cost, which has the potential to reduce public burden on a global scale by intervening in the mental health of individuals [27,28]. Overcoming the shortcomings of traditional face-to-face MHL interventions—specifically, low availability, high threshold for participation, and substantial delivery costs [27,28]—DMHL interventions may be more effective in addressing low levels of MHL in the public [2], which is an impediment to the uptake of, engagement in, and adherence to mental health prevention and intervention [2]. However, research on the effectiveness of DMHL interventions is scattered, and findings on their impact on mental health outcomes are inconclusive. This systematic review and meta-analysis is the first to provide empirical findings on the mental health implications of DMHL interventions.

First, we found that DMHL interventions are as effective as face-to-face interventions in improving MHL (SMD=0.64) and enhancing mental health functioning (SMD=0.42). DMHL interventions greatly improved pretest-posttest proximal DMHL outcomes, which involved various combinations of the 5 DMHL facets—knowledge about obtaining and maintaining good mental health; understanding mental illnesses and treatments; reducing stigma and enhancing help-seeking efficacy and attitudes; and more distal mental health conditions, such as anxiety, depression, loneliness, and distress; and bolstering well-being. Interestingly, we did not find greater uptake of and engagement with DMHL interventions (compared with control conditions), and uptake of DMHL interventions did not moderate the effects on both proximal MHL outcomes and distal mental health outcomes.

Second, our comparison of different DMHL intervention components and conditions, which included *DMHL only* (self-help DMHL psychoeducation) versus waitlist control conditions, *DMHL plus* (DMHL psychoeducation incorporated as a secondary component in other active treatments) versus waitlist control conditions, *DMHL only* versus *DMHL plus*, and *DMHL plus* versus non-DMHL (treatment as usual involving client-professional interactions and therapies such as CBT and dialectical therapy), found differential impacts on mental health outcomes. Compared with waitlist control conditions, *DMHL only* (SMD=0.59) and *DMHL plus* (SMD=0.45; *P*=.02) had similar positive mental health impacts. The effect size of *DMHL only* versus waitlist control was significantly greater than that of *DMHL only* versus *DMHL plus* (SMD=−0.35; *P*=.02), which indicates that the effectiveness of *DMHL only* interventions on mental health was significantly lower than that of *DMHL plus* interventions. On the other hand, the effect size of *DMHL plus* versus waitlist control was similar to that of *DMHL only* versus *DMHL plus* and *DMHL plus* versus non-DMHL (SMD=−0.33; *P*=.02). In other words, compared with waitlist control conditions, *DMHL only*, and non-DMHL, *DMHL plus* had similar positive effects on mental health.

Third, we elucidated whether stronger inferences can be drawn about the intervening effectiveness of DMHL interventions on mental health—whether they are fundamentally effective (pretest-posttest comparison) and more effective than (waitlist) control conditions or other (ie, non-DMHL) interventions. We found larger effect sizes for pretest-posttest DMHL intervention comparisons and waitlist control conditions versus DMHL interventions than for DMHL versus non-DMHL interventions. By comparing immediate and long-term effects, we found the benefits of DMHL interventions to be comparable at postintervention and follow-up assessments, with sustained effects on mental health regardless of longer follow-up assessments. This lack of fade-out of DMHL impacts is important and suggests that being more literate about mental health (recognition, prevention, and management) has long-term benefits for mental well-being. Finally, when considering how individual and contextual characteristics and dissemination methods might moderate the efficacy of DMHL interventions, we found no differences by sex, severity of preexisting mental health conditions, and cultural contexts and only slightly more efficacy in emerging and older adults than in adolescents. Our review and meta-analytic results indicate that digital platform interactivity and dosage of DMHL interventions do not enhance their efficacy. In particular, both new (involving mobile apps, web-based or internet platforms, and social media) and conventional (including films, videos, multimedia, and emails) platforms did not differ significantly in their impacts on mental health. The commonly administered dosage of 10 weeks for digital mental health interventions, including those with DMHL components, demonstrated a similar positive intervening effect on mental health to that of interventions with dosages below and above 10 weeks.

### Implementation and Intervening Effectiveness of DMHL: Uptake and Mental Health

We found that DMHL interventions had a similar impact on mental health to that of traditional face-to-face MHL interventions. Engagement with DMHL interventions, which was assessed as the percentage of users who completed the intervention, ranged from 13.1% to 100% (dropout of 0% to 86.9%). However, participation in DMHL interventions did not differ from that in the control conditions, and uptake of DMHL interventions did not moderate their impact on proximal (DMHL) or distal (mental health) outcomes. Studies of the facilitators of and barriers to the uptake of digital mental health interventions argue that engagement affects intervening effectiveness on mental health outcomes [8,26]. Our review and meta-analysis found limited work on the implementation effectiveness of DMHL interventions involving engagement and uptake that was focused primarily on attrition, indicating a need for future studies to consider other engagement indicators that demonstrate participation in interventions [8,26]. For instance, studies could consider the extent of content accessed (eg, number of modules completed) or engagement in intervention-related activities (eg, number of log-ins or visits, time spent, specific activities or exercises completed, and number of web-based interactions with therapists or peers) [8,26].

Unlike the well-established research on traditional forms of mental health interventions [2], research on digital mental health interventions that leverage technological advancement is nascent [9,41]. Therefore, our findings make important contributions to the growing body of evidence that the implementation and clinical effectiveness of digital mental health interventions are comparable with, if not superior to, those of their traditional face-to-face counterparts [40]. Our study highlights the need for future research to elucidate the development and deployment practices of DMHL interventions for mental health that are scalable and cost-effective and maximize reach [92,93], which can overcome the shortcomings of traditional face-to-face MHL interventions [27,28]. In addition, our meta-analytic results indicate that DMHL interventions have a moderate effect on enhancing mental health outcomes and a strong effect on literacy outcomes. These findings suggest a mechanism of change involving MHL as a mediator or proximal outcome that in turn affects the distal outcomes of mental health conditions [94]. Given that few studies have examined the 5 facets of DMHL conjointly and separately with mental health functioning [16,55], research is needed to elucidate whether and how the 5 facets of DMHL relate to mental health—in particular whether certain facets are essential (or not) or whether combinations of facets yield stronger impacts.

Our meta-analytic efforts to synthesize and compare findings on interventions with different DMHL components revealed aspects of DMHL interventions that were the most promising or effective in bolstering mental health functioning. In particular, *DMHL plus*, which incorporated DMHL as a secondary component with other active treatment components such as skills training; peer support; group discussions and activities; exercises such as diary entries and reflection logs; and informal, nonprofessional counselor interactions [15,21], was the most effective. DMHL plus interventions were superior to waitlist control [15,50] and *DMHL only* conditions [21,51] and comparable with non-DMHL interventions in enhancing mental health functioning [28,52]. Such findings are not surprising given that *DMHL plus* includes some form of active treatment. However, basic forms of DMHL intervention that involve self-help DMHL psychoeducation that builds MHL can be more effective in bolstering mental health than the absence of such interventions [20,21]. This notion was supported by our findings—*DMHL only* interventions that involved self-help DMHL psychoeducation were more effective than waitlist control conditions in enhancing mental health functioning, with large effect sizes. Beyond fundamental effectiveness, in which mental health increases after as compared with before the intervention, DMHL interventions effectively enhanced mental health as compared with waitlist control conditions [21,51]. DMHL interventions had similar effectiveness to that of non-DMHL interventions such as treatment or care as usual that involved professional therapies [28,52].

Our review and meta-analytic results help reconcile the inconsistent findings on the effect of DMHL interventions on mental health documented in the existing literature that resulted from the comparisons of different DMHL components and study designs [15,16]. Although scholars argue that digital mental health interventions, especially DMHL interventions, have the potential to intervene in the mental health of individuals, the absence of consolidated evidence for DMHL interventions’ (clinical) effectiveness presents a roadblock in promoting DMHL as a form of mental health prevention on a global scale [8,9]. Our meta-analytic efforts provide strong support for DMHL interventions achieving mental health effects. DMHL psychoeducation alone or coupled with active treatments is effective in bolstering mental health functioning compared with no intervention. However, for optimal mental health impact, self-help DMHL psychoeducation is not as effective as that provided with other active treatment components, such as skills training; peer support; group discussions and activities; exercises such as diary entries and reflection logs; and informal, nonprofessional counselor interactions. Of note, DMHL interventions that incorporated DMHL into other active treatment components had similar effectiveness to that of treatment as usual and professional therapies (non-DMHL interventions). These findings lend credence to DMHL as a scalable upstream prevention that translates to real-world mental health benefits in bolstering individuals’ mental health functioning [8,9].

Our synthesis of findings across studies on the overall effectiveness of DMHL interventions on mental health, combined with current work demonstrating the ease of access and cost-efficiency of DMHL interventions [27,28], supports the adoption and implementation of DMHL interventions at scale. Such efforts are beneficial at the simple level of self-help DMHL psychoeducation but are even more impactful when coupled with other active treatments. Interventions that combined DMHL with other treatments had the same mental health impact as treatment as usual and professional therapies. Such findings underscore the importance of rigorous intervention designs that examine different components. In particular, using randomized controlled trials (RCTs) to compare and distinguish the impacts of DMHL in combination with different active treatment components such as skills training; peer support; group discussions and activities; exercises such as diary entries and reflection logs; informal, nonprofessional counselor interactions; and care as usual in changing mental health functioning is pivotal. Such a nuanced approach can ascertain how DMHL should be supplemented with active treatment as a necessary and sufficient intervening factor and to establish the relationship to proximal literacy outcomes and distal mental health functioning as mechanisms of change [91].

A key concern regarding digital mental health interventions is their long-lasting impact [36,40]. Particularly for DMHL interventions, the focus on knowledge, beliefs, and attitudes surrounding mental health may render their effectiveness short-lived [46,47]. In contrast, digital mental health interventions such as internet-based CBT are more well established in targeting behavior change and long-term mental health impacts [26]. Our meta-analytic results provide evidence of sustained positive effects of DMHL interventions on mental health. Specifically, studies on DMHL interventions that evaluated immediate postintervention effects and those that assessed carryover effects for as long 34 weeks demonstrated comparable mental health outcomes, particularly in mitigating mental health problems such as depression, anxiety, loneliness, and internalizing and externalizing symptoms [24,25] and bolstering mental well-being, for instance, resilience, life satisfaction, and quality of life [55,95]. More importantly, the sustained effects of DMHL interventions on improved mental health were not attenuated with longer follow-up assessments.

The long-term effects of DMHL interventions on mental health, which averaged 18.2 (SD 3.49; range 4-34) weeks in this synthesis, suggest that the 5 facets of DMHL may be involved in a chain reaction or cascade effect on mental health [94]. Details as to which facets could not be obtained because of a dearth of research that examined all 5 facets of DMHL and mental well-being conjointly and separately. Thus, there are insufficient studies (and number of effect sizes) to determine how the 5 facets of DMHL compare regarding their impacts on mental health. By building knowledge, beliefs, and attitudes regarding mental health, DMHL interventions may have adaptive functions that influence other domains or levels of function (eg, behavior change in treatment uptake and adherence) that spread over time to promote positive mental health development [94]. Future research should unravel the role of the 5 facets of DMHL in well-timed and targeted interventions that promote positive mental health cascades.

### DMHL Intervention Features, Individual Characteristics, and Cultural Contexts as Moderating Factors

The effect of DMHL interventions on mental health holds across new interactive platforms and more conventional, less interactive platforms. Although there are numerous studies examining affordances of digital platforms, such as asynchronicity, anonymity, and social interactions [9], we have a limited understanding of how specific platform affordances interact with mental health—whether they bolster positive mental health or generate and exacerbate mental health problems. Specific to our study, we investigated the interactivity afforded by new and conventional digital platforms in moderating the impact of DMHL interventions on mental health as there are limited studies on DMHL interventions that tap into the full range of digital modalities [9]. Studies investigating social media platforms note different types of affordances that vary across platforms [96]. Importantly, the additive and interactive effects of different affordances in a specific platform could amplify users’ emotional experiences and expressions, resulting in heightened emotional lability and susceptibility to mental health conditions [96]. However, empirical work on whether and how the affordances of social media and other digital platforms affect the effectiveness of digital mental health interventions, including DMHL interventions, is lacking. Future work on DMHL interventions should consider specific platform affordances and their interactions when modulating intervention effectiveness.

Unexpectedly, our synthesis did not find dosage effect. Although other studies have found the median dosage or duration of interventions to be approximately 10 weeks [60], our meta-regressions indicated that DMHL interventions with a dosage of 10 weeks had similar impacts on enhancing mental health as dosages below and above 10 weeks. Our amalgamated findings suggest that the 10-week dosage that is commonly adopted by digital mental health interventions may not be optimal for DMHL interventions [61,62]. More research involving RCTs on digital mental health and DMHL interventions alike that examine the effects of varying dosages of interventions on mental health outcomes is warranted. However, our findings are promising given the challenges with mental health services and MHL intervention retention found in many countries [97,98]. Collectively, our meta-analysis and meta-regressions on the moderating role of DMHL intervention features provide important insights into best practices in DMHL that enhance mental health. Consistent with global efforts to raise mental health awareness and improve poor MHL in the public [1], this synthesis advances our understanding of DMHL by building a conceptual model of MHL [4] that could inform the design and implementation of evidence-based DMHL interventions [8].

DMHL intervention effects in this synthesis were comparable across sex and severity of mental health conditions. Other studies have found that female participants have higher levels of MHL [64,65], which can moderate the effects of DMHL interventions on mental health outcomes. When controlling for pre- and postintervention levels of MHL, we found that DMHL interventions had equivalent effects on increasing the mental health functioning of both male and female participants. Contrary to findings on the reduced effectiveness of digital mental health interventions in individuals with mental illness or with physical ill health [8], we found that the severity of mental health conditions did not attenuate the effectiveness of DMHL interventions on the mental health of healthy populations. We expected DMHL interventions to demonstrate greater effects on adolescents than on emerging and older adults given that adolescents are the primary users of digital platforms such as social media, mobile apps, and web-based mental health resources [30]. However, DMHL interventions appear to be most effective in emerging and older adults. The conceptual model of MHL describes the 5 facets of DMHL as positively associated with maturity, cognitive advancement, and educational attainment in adulthood [4]. As such, this helps explain why DMHL interventions had a greater impact on emerging and older adults’ mental health. With increasing mental health concerns in adolescence [32,33], future research needs to illuminate whether and how some facets of DMHL are applicable to bolstering adolescent mental health and target these facets in the design and implementation of DMHL interventions for youth mental health.

In contrast to our hypothesis, the impact of DMHL interventions did not differ between Western and Eastern cultural contexts. Most Asian societies with collectivistic cultural characteristics are conservative in the way in which they address mental health concerns and often promote MHL and positive mental health functioning in ways that differ from those of Western cultural contexts with individualistic cultural characteristics. For instance, cultivating interdependence relations, relational harmony, and dialectical beliefs and emotions may be more common in Eastern cultures [68]. Furthermore, the Asian community as a whole, including policy makers, mental health professionals, and the public, is only beginning to embrace the notion of digital mental health interventions [99,100]. On the other hand, studies on digital mental health interventions, especially DMHL interventions, are conducted predominantly in Western cultural contexts in high-income countries (Global North) [8,9], where there is greater public awareness of and less stigma associated with mental health conditions and greater focus on developing a healthy sense of independent self, including positive mental health [67,68].

Consistent with most mental health research [42-44], Western cultural contexts were overrepresented in the DMHL interventions, particularly countries in the Global North such as Australia, the United States, and the United Kingdom. Comparatively, there are limited studies conducted in the Global South that involve Eastern cultural contexts in Asian countries and scant research on DMHL interventions from South American and African countries, limiting assessments of DMHL intervention effectiveness in the Global South. Furthermore, most studies on DMHL interventions included in this review did not provide information on the ethnic or racial composition of their samples, and those that provided this information had samples that were predominantly White with limited racial or ethnic diversity. Our findings reinforce existing research on digital mental health interventions broadly that indicated that these interventions were designed for and evaluated on implementation and clinical effectiveness with largely homogeneous samples that are disproportionately White, from the Global North, and likely cissex [42-44]. Findings from our review and meta-analysis on DMHL, coupled with those on digital mental health interventions [42-44], highlight the need for more consideration of diversity in research but broadly support the utility of DMHL interventions.

In general, there is lower MHL, greater mental health stigma, and fewer services provided in the Global South [42-44]. DMHL interventions may be an important and accessible mental health resource that could not only provide greater access and reach and reduce stigma but also improve MHL among the public [42-44]. Our review found that DMHL interventions are implemented and evaluated mainly in Western cultural contexts in high-income countries [42-44] and some contemporary Asian societies [99,100]. Our results call for greater research attention to the design, implementation, and evaluation of culturally appropriate DMHL interventions in Asian, South American, and African countries, as well as more diversity in research in the Global North. Contextual stressors associated with racial and ethnic marginalization and mental health disparities, including lower access to and greater barriers to engaging in digital mental health interventions, underscore the need for future work on DMHL interventions specifically and digital mental health interventions broadly that target racial or ethnic minority groups [42-44].

### Limitations

Our systematic review and meta-analysis highlights 4 major limitations in the broader literature on DMHL interventions. First, there is a lack of a clear conceptualization that distinguishes the DMHL components assessed in the interventions. Our review of existing literature and efforts to synthesize findings on the effectiveness of DMHL interventions suggest that, conceptually, *DMHL only* interventions refer to those that implement DMHL as a self-help psychoeducation component. In contrast, *DMHL plus* interventions incorporate DMHL as a secondary component with other active treatment components that are nonprofessional and informal in nature, for instance, skills training; peer support; group discussions and activities; exercises such as diary entries and reflection logs; and informal counselor interactions, and non-DMHL interventions refer to treatment as usual that involves client-professional visits, interactions, and therapies. Although receiving professional mental health treatment likely improves knowledge about mental health symptoms and management (eg, MHL), such interventions are not specifically focused on literacy. As such, they are categorized as non-DMHL interventions. These conceptual distinctions in DMHL interventional components have yet to be acknowledged in the current research despite the fact that all DMHL interventions included in our meta-analysis examined various conditions that could be classified as *DMHL only*, *DMHL plus*, and non-DMHL.

Second, DMHL interventions are not always clear about the facet of DMHL examined and rarely refer to the theoretical framework of DMHL that distinguishes five literacy facets [5,6]: (1) knowledge about obtaining and maintaining good mental health, (2) understanding mental illnesses and treatments, (3) reducing mental illness–related stigma, (4) enhancing help-seeking efficacy or behaviors, and (5) enhancing help-seeking attitudes or intentions. We recommend that future research recognize the conceptualization of DMHL facets and be clear about the facet of DMHL that demonstrates a positive relationship to or efficacy in enhancing mental health. Such an approach would facilitate a comprehensive and converged understanding of the conditions under which DMHL most strongly relates to and effectively increases mental health functioning. On a similar note, DMHL interventions do not always include both proximal literacy outcomes and distal mental health outcomes, which are important in establishing mechanisms of change with proximal literacy outcomes as possible mediators. As the field of DMHL interventions is nascent [8,9], studies have primarily focused on examining mental health outcomes (clinical outcomes) [8,9], and a major gap remains in understanding implementation effectiveness involving uptake and engagement. Drawing from work on digital mental health interventions, user uptake and engagement can vary across different indicators and affect intervening effectiveness on mental health outcomes [8,26]. Extant research on digital mental health interventions, including DMHL, largely focuses on attrition as an indicator of uptake and engagement. Future DMHL interventions should consider other engagement indicators to demonstrate the extent of intervention use and potential key features for effectiveness.

Third, the features of DMHL interventions, such as dosage and platform affordances, warrant greater attention given that research on digital mental health interventions has demonstrated how these features can influence user engagement and the effectiveness of the intervention [8,9]. This synthesis was limited to sex comparisons involving cissex individuals even though transsex and nonbinary individuals are at greater risk of mental health issues. Future work needs to expand the consideration of sex beyond binary operationalizations. We did not find evidence for the role of the commonly endorsed dosage of 10 weeks of digital mental health interventions that included DMHL components and of platform interactivity affordances in amplifying the mental health impact of DMHL interventions. Future work should examine varying dosages of DMHL interventions and different affordances and their additive and interactive effects through empirical examination using an RCT study design.

Finally, most DMHL interventions (56/76, 74%) were found in Western cultural contexts, especially in high-income countries (Global North), and most studies on DMHL interventions (36/76, 47%) did not report information on the racial or ethnic composition of their samples. Among those that did, the samples were predominantly White with limited racial or ethnic diversity. As such, our findings may not be generalizable to other geographic regions or demographic groups. In light of contextual stressors associated with racial and ethnic marginalization and mental health disparities, future work on DMHL interventions that target racial or ethnic minority groups, particularly the design, adoption, and evaluation of the effects of culturally adaptive DMHL interventions on uptake and mental health functioning, is needed [42-44]. Collectively, our review and meta-analysis of DMHL and mental health makes important theoretical and practical contributions. For theory building on DMHL, we found evidence for the fundamental effectiveness of DMHL interventions that increased postintervention mental health and strong inferences for DMHL interventions’ effectiveness in bolstering mental health as compared with waitlist control conditions. However, DMHL interventions confer optimal effects on mental health when DMHL psychoeducation is incorporated with informal, nonprofessional active treatment components such as skills training and peer support, which demonstrates comparable effectiveness with that of treatment as usual involving client-professional interactions and therapies. However, none of the interventions in this review considered the mechanism of action regarding how DMHL affects proximal outcomes that in turn affect distal mental health conditions. The large effect size of DMHL interventions on MHL outcomes indicates that literacy may serve as a mediating mechanism for enhancing mental health functioning. Thus, the MHL framework needs to unpack and incorporate different components of DMHL, especially in how they relate to the 5 DMHL facets, and consider their interplay with various active treatment components. More importantly, proximal and distal factors involved in the mechanism of action of DMHL and mental health need to be examined to build and expand the MHL framework.

### Conclusions

Our review and meta-analysis found that DMHL interventions are as effective as face-to-face interventions. Basic DMHL interventions with self-help DMHL psychoeducation had similar effectiveness to that of interventions that incorporated DMHL as a secondary component with other active treatment components in bolstering mental health functioning. These findings are practically meaningful and underscore the feasibility and promise of digital modalities for improving mental health. DMHL interventions greatly increased literacy outcomes and moderately improved mental health functioning by reducing depression, anxiety, loneliness, and internalizing and externalizing symptoms and enhancing quality of life and resilience. Importantly, these effects, which did not differ by platform type or dosage, were sustained over time. Future research is needed to test our findings on the circumstances in which DMHL interventions are the most effective in enhancing mental health—specific DMHL components, dosage, extent of carryover effects, platform affordances, and individual and contextual factors—to aid policy makers, mental health professionals, and social services in establishing high-performance DMHL interventions that enhance mental health in the community.

## Appendix

Multimedia Appendix 1 Full search strings.

Multimedia Appendix 2 Characteristics of the included digital mental health literacy interventions and their traditional face-to-face counterparts.

Multimedia Appendix 3 Summary and sample characteristics of the digital mental health literacy interventions.

Multimedia Appendix 4 Forest plot of data investigating the effects of digital mental health literacy and traditional face-to-face mental health literacy interventions on mental health.

Multimedia Appendix 5 Funnel plot of the effect sizes of the digital mental health literacy and traditional face-to-face mental health literacy interventions on mental health.

Multimedia Appendix 6 Moderators of digital mental health literacy interventions and mental health.

Multimedia Appendix 7 PRISMA 2020 checklist.

## Abbreviations

CBT: cognitive behavioral therapy
DMHL: digital mental health literacy
MHL: mental health literacy
PRISMA: Preferred Reporting Items for Systematic Reviews and Meta-Analyses
RCT: randomized controlled trial
RQ: research question
SMD: standardized mean difference

## Data Availability

The data sets generated during and/or analyzed during this study are available on the Open Science Framework [101].
