# Supplementary material for: The Effect of Digital Mental Health Literacy Interventions on Mental Health: Systematic Review and Meta-Analysis
Source: J Med Internet Res. 2024 Feb 29;26:e51268. doi: 10.2196/51268 (PMC10941000; doi:10.2196/51268)
Supplement: Multimedia Appendix 1 [file jmir_v26i1e51268_app1.docx]

Multimedia Appendix 1.

Table S1. Three search strategies

|  | First, a systematic search was conducted in September 2022 across PsycINFO, PubMed, EMBASE, and CINAHL because these databases cover two fields pertinent to this review—social science and medical research. Full and truncated search terms were included for title, abstract, and keywords. |
| --- | --- |
|  | Second, reference lists of the included studies (including other reviews and meta-analyses) were searched manually and cross-referenced for additional articles. |
|  | Searches were re-run just before the final analyses in May 2023 to include further articles published from the inceptions of the four databases. Limits, when available, were applied to include only academic articles, human studies, and papers written in English or with an English translation. Together, these search methods identified 42,014 citations to relevant studies. |

Table S2. Search terms used for the formulation of the search strings in the systematic search

| **PICO concept** | **Search Terms** |
| --- | --- |
| Population | Individuals learning mental health literacy or engaging in traditional and/or digital mental health resources |
| Intervention (or Exposure) | Digital mental health literacy; traditional mental health literacy |
| Comparison | Waitlist control, care/treatment as usual, alternative interventions |
| Outcome | Mental well-being, psychological well-being, psychological functioning, psychosocial functioning, psychosocial well-being, mental health (e.g., depression, anxiety, internalizing and externalizing symptoms) |
| Inclusion and exclusion criteria were established *a priori* | |
| Inclusion criteria | (a) Be a full-text empirical study written in English;  (b) Provide sufficient statistics for calculation of effect sizes. Field studies needed to supply correlation coefficients; interventions needed to list sample sizes, means, and standard deviations (SDs). For studies that lacked statistical information needed to calculate effect sizes, authors were contacted to provide the missing data;  (c) Field studies—cross-sectional and longitudinal—needed to include at least one facet of mental health literacy (i.e., knowledge about obtaining and maintaining good mental health; understanding mental illnesses and treatments; reducing mental illness-related stigma; enhancing help-seeking efficacy/behaviors; help-seeking attitudes/intentions; [4]);  (d) Interventions needed to have at least one facet of mental health literacy (i.e., knowledge about obtaining and maintaining good mental health; understanding mental illnesses and treatments; reducing mental illness-related stigma; enhancing help-seeking efficacy/behaviors; help-seeking attitudes/intentions; [4]);  (e) Include at least one measure of mental health or well-being (refer to outcomes above under PICO); |
| Exclusion criteria | a) No experimental or quasi-experimental study designs and no relevant statistics for the calculation of effect sizes (e.g., qualitative studies, theory or protocol papers for intervention; 29 articles);  (b) Wrong population (e.g., assessing effect of mental health literacy on trainers; 32 articles);  (c) Wrong intervention (e.g., assessing therapeutic modalities or mental health screening and monitoring with no mental health literacy intervention or measure; 105 articles);  (d) Wrong outcomes (e.g., assessing outcomes such as feasibility without proximal DMHL outcomes and/or distal mental health outcomes such as depression or anxiety; 55 articles);  (e) No English full-text available (55 articles) |

Table S3. Full search strings

| Database | Search String | Number of outcomes |
| --- | --- | --- |
| Ovid  APA PsycInfo [Coverage: 1806 to September Week 4 2023] | ('mental health literacy' or 'mental health knowledge' or 'mental health training' or 'mental health curriculum' or 'mental health education').ab, ti, sh.  ('mental health AND literacy' or 'mental health AND knowledge' or 'mental health AND training' or 'mental health AND curriculum' or 'mental AND health AND education').ab, ti, sh, id.  ('digital mental health literacy' or 'digital mental health intervention' or 'digital mental health knowledge' or 'digital mental health training' or 'digital mental health curriculum' or 'digital mental health education' or ‘social media mental health literacy’).ab, ti, sh, id.  ('digital AND literacy AND mental health’ or ‘digital AND mental health AND intervention’ or 'digital AND mental health AND knowledge' or 'digital AND mental health AND training' or 'digital AND mental health AND curriculum' or 'digital AND mental health AND education' or 'social media AND literacy AND mental health'). ab, ti, sh, id.  (('digital' or 'eHealth' or 'web-based' or 'internet-based' or 'mobile' or 'gamification' or 'forums' or 'social media' or 'social networking sites' or 'Discord' or 'Facebook' or 'Instagram' or 'Reddit' or 'Snapchat' or 'Telegram' or 'TikTok' or 'Twitch' or 'Twitter' or 'WeChat' or 'WhatsApp' or 'YouTube' or 'online' or 'website' or 'app' or 'virtual' or 'video' or 'podcast' or 'interactive tutorial' or 'discussion boar' or 'video games').  AND  ('literacy' or 'knowledge' or 'training' or 'curriculum' or 'education' or 'intervention').  AND  ('mental health' or 'mental wellness' or 'mental well-being' or 'psychological well-being' or 'psychological functioning' or 'psychosocial functioning' or 'psychosocial well-being' or 'anxiety' or 'depression' or 'internalizing symptoms' or 'externalizing symptoms' or 'mental health disorders' or 'burnout’ or 'eating disorder')). ab, ti, sh, id | 5628 |
| PubMed  [no subject heading and (author assigned) keyword fields; mesh subheadings retrieved 0 results  Coverage: 2013-2023] | ("mental health literacy"[Title/Abstract] OR "mental health knowledge"[Title/Abstract] OR "mental health training"[Title/Abstract] OR "mental health curriculum"[Title/Abstract] OR "mental health education"[Title/Abstract] OR "digital mental health literacy"[Title/Abstract] OR "digital mental health intervention"[Title/Abstract] OR 'digital mental health knowledge” [Title/Abstract] OR 'digital mental health training' [Title/Abstract] OR 'digital mental health curriculum' [Title/Abstract] OR 'digital mental health education' [Title/Abstract] OR ‘social media mental health literacy’[Title/Abstract]  (("mental health [Title/Abstract] AND literacy"[Title/Abstract] OR "mental health [Title/Abstract] AND knowledge"[Title/Abstract] OR "mental health [Title/Abstract] AND training"[Title/Abstract] OR "mental health[Title/Abstract] AND curriculum" [Title/Abstract] OR "mental health [Title/Abstract] AND education"[Title/Abstract] OR“digital [Title/Abstract] AND literacy [Title/Abstract] AND mental health[Title/Abstract]“ OR “digital [Title/Abstract] AND mental health [Title/Abstract] AND intervention [Title/Abstract]“ OR “digital [Title/Abstract] AND mental health [Title/Abstract] AND knowledge [Title/Abstract]“ OR “digital [Title/Abstract] AND mental health [Title/Abstract] AND training[Title/Abstract]“ OR “digital [Title/Abstract] AND mental health [Title/Abstract] AND curriculum [Title/Abstract]“ OR “digital [Title/Abstract] AND mental health [Title/Abstract] AND education [Title/Abstract]“ OR “social media[Title/Abstract] AND literacy [Title/Abstract] AND mental health [Title/Abstract]“  (("digital"[Title/Abstract] OR "eHealth"[Title/Abstract] OR "web-based"[Title/Abstract] OR "internet-based"[Title/Abstract] OR "mobile"[Title/Abstract] OR "gamification"[Title/Abstract] OR "forums"[Title/Abstract]) OR “online“ [Title/Abstract] OR “website“ [Title/Abstract] OR “app“ [Title/Abstract] OR “virtual“ [Title/Abstract] OR “video“[Title/Abstract] OR “podcast“ [Title/Abstract] OR “interactive tutorial“ [Title/Abstract] OR “discussion board“ [Title/Abstract] OR “video games“ [Title/Abstract] AND ("literacy"[Title/Abstract] OR "knowledge"[Title/Abstract] OR "training"[Title/Abstract] OR "curriculum"[Title/Abstract] OR "education"[Title/Abstract] OR "intervention"[Title/Abstract]) AND ("mental health"[Title/Abstract] OR "mental wellness"[Title/Abstract] OR "mental well-being"[Title/Abstract] OR "psychological well-being"[Title/Abstract] OR "psychological functioning"[Title/Abstract] OR "psychosocial functioning"[Title/Abstract] OR "psychosocial well-being"[Title/Abstract] OR "psychosocial well-being"[Title/Abstract] OR “anxiety“[Title/Abstract] OR “depression“[Title/Abstract] OR “internalizing symptoms“[Title/Abstract] OR “externalizing symptoms“[Title/Abstract] OR “mental health disorders“ [Title/Abstract] OR ''eating disorder''[Title/Abstract] OR ''burnout''[Title/Abstract]))  (("social media"[Title/Abstract] OR "social networking sites"[Title/Abstract] OR"Discord"[Title/Abstract] OR "Facebook"[Title/Abstract] OR "Instagram"[Title/Abstract] OR "Reddit"[Title/Abstract] OR "Snapchat"[Title/Abstract] OR "Telegram"[Title/Abstract] OR "TikTok"[Title/Abstract] OR "Twitch"[Title/Abstract] OR "Twitter"[Title/Abstract] OR "WeChat"[Title/Abstract] OR "WhatsApp"[Title/Abstract] OR "YouTube"[Title/Abstract]) AND ("literacy"[Title/Abstract] OR "knowledge"[Title/Abstract] OR "training"[Title/Abstract] OR "curriculum"[Title/Abstract] OR "education"[Title/Abstract] OR "intervention"[Title/Abstract]) AND ("mental health"[Title/Abstract] OR "mental wellness"[Title/Abstract] OR "mental well-being"[Title/Abstract] OR "psychological well-being"[Title/Abstract] OR "psychological functioning"[Title/Abstract] OR "psychosocial functioning"[Title/Abstract] OR "psychosocial well-being"[Title/Abstract] OR “anxiety“ [Title/Abstract] OR “depression“ [Title/Abstract] OR “internalizing symptoms“ [Title/Abstract] OR “externalizing symptoms“ [Title/Abstract] OR “mental health disorders“ [Title/Abstract] OR ''burnout''[Title/Abstract] OR ''eating disorder''[Title/Abstract])) | 7777 |
| Embase  (Embase.com)  [Coverage: 2013 to 2023] | ('mental health literacy':ab,ti,kw OR 'mental health knowledge':ab,ti,kw OR 'mental health training':ab,ti,kw OR 'mental health curriculum':ab,ti,kw OR 'mental health education':ab,ti,kw OR 'digital mental health literacy':ab,ti,kw OR 'digital mental health intervention':ab,ti,kw)  ('mental health:ab,ti AND literacy':ab,ti,kw OR 'mental health:ab,ti,kw AND knowledge':ab,ti,kw OR 'mental health:ab,ti,kw AND training':ab,ti,kw OR 'mental health:ab,ti,kw AND curriculum':ab,ti,kw OR 'mental health:ab,ti,kw AND education':ab,ti,kw OR ‘digital:ab,ti,kw AND literacy:ab,ti,kw AND mental health‘:ab,ti,kw OR ‘digital:ab,ti,kw AND mental health:ab,ti,kw AND intervention‘:ab,ti,kw OR ‘digital:ab,ti,kw AND mental health:ab,ti,kw AND knowledge‘:ab,ti,kw or ‘digital:ab,ti,kw AND mental health:ab,ti,kw AND training‘:ab,ti,kw OR ‘digital:ab,ti,kw AND mental health:ab,ti,kw AND curriculum‘:ab,ti,kw OR ‘digital:ab,ti,kw AND mental health:ab,ti,kw AND education‘:ab,ti,kw OR ‘social media:ab,ti,kw AND literacy:ab,ti,kw AND mental health‘:ab,ti,kw  [this search string does not apply to Embase]  (('digital':ab,ti,kw OR 'ehealth':ab,ti,kw OR 'web-based':ab,ti,kw OR 'internet-based':ab,ti,kw OR 'mobile':ab,ti,kw OR gamification:ab,ti,kw OR 'forums':ab,ti,kw) OR ‘Online‘:ab,ti,kw OR ‘website‘:ab,ti,kw OR ‘app‘:ab,ti,kw OR ‘virtual‘:ab,ti,kw OR ‘video‘:ab,ti,kw OR ‘podcast‘: ab,ti,kw OR ‘interactive tutorial‘:ab,ti,kw OR ‘discussion board‘:ab,ti,kw OR ‘video games‘:ab,ti,kw) AND ('literacy':ab,ti,kw OR 'knowledge':ab,ti,kw OR 'training':ab,ti,kw OR 'curriculum':ab,ti,kw OR 'education':ab,ti,kw OR 'intervention':ab,ti,kw) AND ('mental health':ab,ti,kw OR 'mental wellness':ab,ti,kw OR 'mental well-being':ab,ti,kw OR 'psychological well-being':ab,ti,kw OR 'psychological functioning':ab,ti,kw OR 'psychosocial functioning':ab,ti,kw OR 'psychosocial well-being':ab,ti,kw OR ‘anxiety‘:ab,ti,kw OR ‘depression‘:ab,ti,kw OR ‘internalizing symptoms‘:ab,ti,kw OR ‘externalizing symptoms‘:ab,ti,kw OR ‘mental health disorders’:ab,ti,kw OR 'burnout':ab,ti,kw OR 'eating disorder':ab,ti,kw))  (('social media':ab,ti,kw OR 'social networking sites':ab,ti,kw OR 'discord':ab,ti,kw OR 'facebook':ab,ti,kw OR instagram:ab,ti,kw OR 'reddit':ab,ti,kw OR 'snapchat':ab,ti,kw OR 'telegram':ab,ti.kw OR 'tiktok':ab,ti,kw OR 'twitch':ab,ti.kw OR 'twitter':ab,ti,kw OR 'wechat':ab,ti,kw OR 'whatsapp':ab,ti,kw OR 'youtube':ab,ti,kw) AND ('literacy':ab,ti,kw OR 'knowledge':ab,ti,kw OR 'training':ab,ti,kw OR 'curriculum':ab,ti,kw OR 'education':ab,ti,kw OR 'intervention':ab,ti,kw) AND ('mental health':ab,ti,kw OR 'mental wellness':ab,ti,kw OR 'mental well-being':ab,ti,kw OR 'psychological well-being':ab,ti,kw OR 'psychological functioning':ab,ti,kw OR 'psychosocial functioning':ab,ti,kw OR 'psychosocial well-being':ab,ti,kw OR ‘anxiety‘:ab,ti,kw OR ‘depression‘:ab,ti,kw OR ‘internalizing symptoms‘:ab,ti,kw OR ‘externalizing symptoms‘:ab,ti,kw OR ‘mental health disorders’:ab,ti,kw OR 'burnout':ab,ti,kw OR 'eating disorder':ab,ti,kw)) | 10,951 |
| CINAHL complete  [Coverage: 1954 to 2023] | (TI (“mental health literacy” OR “mental health knowledge” OR “mental health training” OR “mental health curriculum” OR “mental health education” OR “digital mental health literacy” OR “digital mental health intervention” ) OR AB ( “mental health literacy” OR “mental health knowledge” OR “mental health training” OR “mental health curriculum” OR “mental health education” OR “digital mental health literacy” OR “digital mental health intervention” ) OR MW (“mental health literacy” OR “mental health knowledge” OR “mental health training” OR “mental health curriculum” OR “mental health education” OR “digital mental health literacy” OR “digital mental health intervention”))  (TI (“mental health AND literacy” OR “mental health AND knowledge” OR “mental health AND training” OR “mental health AND curriculum” OR “mental health AND education” OR “digital AND mental health AND literacy” OR “digital AND mental health AND intervention” ) OR AB ( “mental health AND literacy” OR “mental health AND knowledge” OR “mental health AND training” OR “mental health AND curriculum” OR “mental health AND education” OR “digital AND mental health AND literacy” OR “digital AND mental health AND intervention” ) OR MW (“mental health AND literacy” OR “mental health AND knowledge” OR “mental health AND training” OR “mental health AND curriculum” OR “mental health AND education” OR “digital AND mental health AND literacy” OR “digital AND mental health AND intervention”))  (TI (“digital” OR “eHealth” OR “web-based” OR “internet-based” OR “mobile” OR ”gamification” OR “forums” OR “Online” OR “website” OR “app”OR “virtual” OR “video” OR “podcast” OR “interactive tutorial” OR “discussion board” OR “video games”) OR AB ( “digital” OR “eHealth” OR “web-based” OR “internet-based” OR “mobile” OR ”gamification” OR “forums” OR “Online” OR “website” OR “app” OR “virtual” OR “video” OR “podcast” OR “interactive tutorial” OR “discussion board” OR “video games”) OR MW(“digital” OR “eHealth” OR “web-based” OR “internet-based” OR “mobile” OR ”gamification” OR “forums” OR “Online” OR “website” OR “app” OR “virtual” OR “video” OR “podcast” OR “interactive tutorial” OR “discussion board” OR “video games”))  AND  (TI (“literacy” OR “knowledge” OR “training” OR “curriculum” OR “education” OR “intervention” ) OR AB ( “literacy” OR “knowledge” OR “training” OR “curriculum” OR “education” OR “intervention”) OR MW (“literacy” OR “knowledge” OR “training” OR “curriculum” OR “education” OR “intervention”))  AND  (TI (“mental health” OR “mental wellness” OR “mental well-being” OR “psychological well-being” OR “psychological functioning” OR “psychosocial functioning” OR “psychosocial well-being” OR “anxiety” OR “depression” OR “internalizing symptoms” OR “externalizing symptoms” OR “mental health disorders” OR “burnout” OR “eating disorder”) OR AB (“mental health” OR “mental wellness” OR “mental well-being” OR “psychological well-being” OR “psychological functioning” OR “psychosocial functioning” OR “psychosocial well-being” OR “anxiety” OR “depression” OR “internalizing symptoms” OR “externalizing symptoms” OR “mental health disorders” OR “burnout” OR “eating disorder”) OR MW (“mental health” OR “mental wellness” OR “mental well-being” OR “psychological well-being” OR “psychological functioning” OR “psychosocial functioning” OR “psychosocial well-being” OR “anxiety” OR “depression” OR internalizing symptoms” OR “externalizing symptoms” OR “mental health disorders” OR “burnout” OR “eating disorder”))  (TI ( “social media” OR “social networking sites” OR “Discord” OR “Facebook” OR ”Instagram” OR “Reddit” OR “Snapchat” OR “Telegram” OR “TikTok” OR “Twitch” OR “Twitter” OR “WeChat” OR “WhatsApp” OR “YouTube” ) OR AB ( “social media” OR “social networking sites” OR “Discord” OR “Facebook” OR ”Instagram” OR “Reddit” OR “Snapchat” OR “Telegram” OR “TikTok” OR “Twitch” OR “Twitter” OR “WeChat” OR “WhatsApp” OR “YouTube” ) OR MW (“social media” OR “social networking sites” OR “Discord” OR “Facebook” OR ”Instagram” OR “Reddit” OR “Snapchat” OR “Telegram” OR “TikTok” OR “Twitch” OR “Twitter” OR “WeChat” OR “WhatsApp” OR “YouTube”))  AND  (TI (“literacy” OR “knowledge” OR “training” OR “curriculum” OR “education” OR “intervention” ) OR AB (“literacy” OR “knowledge” OR “training” OR “curriculum” OR “education” OR “intervention”) OR MW (“literacy” OR “knowledge” OR “training” OR “curriculum” OR “education” OR “intervention”))  AND (TI (“mental health” OR “mental wellness” OR “mental well-being” OR “psychological well-being” OR “psychological functioning” OR “psychosocial functioning” OR “psychosocial well-being” ) OR AB ( “mental health” OR “mental wellness” OR “mental well-being” OR “psychological well-being” OR “psychological functioning” OR “psychosocial functioning” OR “psychosocial well-being” OR “anxiety” OR “depression” OR “internalizing symptoms” OR “externalizing symptoms” OR “mental health disorders” OR “burnout” OR “ eating disorder”)) | 17658 |
| Total | | 42,014 |

Note. Across all four databases, truncated terms were added with*
