# Supplementary material for: The Effect of Digital Mental Health Literacy Interventions on Mental Health: Systematic Review and Meta-Analysis
Source: J Med Internet Res. 2024 Feb 29;26:e51268. doi: 10.2196/51268 (PMC10941000; doi:10.2196/51268)
Supplement: Multimedia Appendix 2 [file jmir_v26i1e51268_app2.docx]

Multimedia Appendix 2.

Table S1. Study characteristics on DMHL interventions and traditional, face-to-face mental health literacy interventions

| **No.** | **Author(s)** | **Year** | **N^a^** | **M_age_** | **Females** | **Culture^b^** | **Mode^d^** | **Intervention condition^e^** | **DMHL component^f^** | **Duration^g^** | **Follow-up^h^** | **% uptake of Control and (Intervention)^i,j,k^** | **Ethnic composition** |
| --- | --- | --- | --- | --- | --- | --- | --- | --- | --- | --- | --- | --- | --- |
| 1 | Ali et al. | 2014 | 241 | 20.5 | 69 | W | 2 | 3 | 3 | 32 | 32 | 87.5  (96)^i^ | No ethnic breakdown |
| 2 | Alvarez-Jimenez et al. | 2021 | 170 | 20.91 | 47.1 | W | 2 | 2 | 4 | 18 | 12,18 | 75  (73.3)^i^ | No ethnic breakdown |
| 3 | Andrews et al. | 2022 | 2539 | 13.6 | NIL | W | 2 | 2 | 4 | 12 | 12,24,48 | 70.4  (59.4)^j^ | No ethnic breakdown |
| 4 | Arjadi et al. | 2018 | 313 | 24.5 | 81 | E | 2 | 3 | 3 | 10 | 12,24 | 70.4  (93.5)^i^ | 42% Java  12% Tionghoa  14% Sunda  10% Batak  4% Minangkabau  19%Other (19 ethnicities) |
| 5 | Bakker et al. | 2018 | 226 | 34 | 81 | W | 2 | 2 | 2 | 4 | NIL | 67.9  (50)^i^ | No ethnic breakdown |
| 6 | Baumeister et al. | 2021 | 210 | 49.9 | 60 | W | 2 | 2 | 4 | 9 | 15 | 75.20  (84.6)^i^ | No ethnic breakdown |
| 7 | Bjornsen et al. | 2018 | 357 | 15.6 | 53 | W | 1 | NIL | NIL | 12 | NIL | 93.5  (100) | No ethnic breakdown |
| 8 | Blaney et al. | 2021 | 28 | 58.4 | 85.7 | W | 2 | 1 | 2 | 12 | 3,6 | NIL  (97.1)^i^ | No ethnic breakdown |
| 9 | Booth et al. | 2022 | 536 | 13.47 | 62.87 | W | 1 | NIL | NIL | 1 | NIL | NIL  (NIL)^j^ | No ethnic breakdown |
| 10 | Carl et al. | 2020 | 256 | 30.9 | 70 | W | 2 | 2 | 2 | 6 | 6,16 | 96.9  (80.5)^i^ | 0% Arab  2% Asian  4% Black  6% Mixed  2%Other  85% White  1% Did not state |
| 11 | Carr et al. | 2018 | 60 | NIL | 68.3 | W | 1 | 1 | NIL | 1 | NIL | NIL  (98.3)^j^ | No ethnic breakdown |
| 12 | Castro et al. | 2012 | 1645 | NIL | 3.8 | W | 1 | NIL | NIL | 24 | NIL | NIL  (36.1)^i^ | No ethnic breakdown |
| 13 | Cernvall et al. | 2018 | 11 | 38.6 | 73 | W | 2 | 1 | 2 | 4 | NIL | NIL  (33.3)^k^ | No ethnic breakdown |
| 14 | Chrisholm et al. | 2016 | 657 | 12.21 | 52.05 | W | 1 | NIL | NIL | 2 | 24 | 87.4  (83.2)^j^ | 28.5% South Asians; 52.9 White;  8.92 black;  9.68 others |
| 15 | Cieslak et al. | 2016 | 168 | 37.49 | 78 | W | 2 | 3 | 3 | 8 | 8 | 90.8  (92.6)^i^ | No ethnic breakdown |
| 16 | Clough et al. | 2020 | 45 | 25.8 | 60 | W | 1 | NIL | NIL | 1 | NIL | 100  (100) | 51%Asian;  12% black;  19%White;  12%South American;  8% others |
| 17 | De Silva | 2022 | 176 | 24.4 | 80.7 | W | 2 | 2 | 1 | 1 | 3 | 77.2  (79.1)^k^ | Latinx-Oriented to Approximately Balanced (56.8%); Slightly Anglo  Oriented/Bicultural (29.6%);  Very Latinx Oriented (6.3%) Very Anglo Oriented (4.0%). |
| 18 | DeLuca | 2021 | 206 | 15.4 | 56.2 | W | 1 | NIL | NIL | 8 | 8 | 83.2  (82.9)^j^ | No ethnic breakdown |
| 19 | Ebert et al. | 2021 | 396 | 41.76 | 76.3 | W | 2 | 2 | 4 | 7 | 24 | 90.4  (67.7) | 80.6% Caucasian/White  2.3% Asian  0.5% Hispanic  16.7% Prefer not to say |
| 20 | Ebert et al. | 2016 | 264 | 42 | 72 | W | 2 | 2 | 2 | 7 | 24 | 98  (83)^i^ | 83% white;  1% Asian;  43% others |
| 21 | Espie et al. | 2019 | 1711 | 48 | 77.7 | W | 2 | 3 | 4 | 8 | 8,24 | 57.7  (48).2^i^ | 2.5% Asian  0.8% Black  2.3% Mixed  1.4% Other  92.0% White  0.9% Do not wish to state |
| 22 | Eustis et al. | 2018 | 156 | 25.4 | 78.8 | W | 2 | 2 | 2 | 4 | 8 | 44.9  (37.2)^j^ | 1.3% Native American;  22.4% Asian;  15% black;  14.1% Latino;  1.5% North American;  54.5% White;  7.1% multiracial |
| 23 | Fogarty et al. | 2017 | 144 | 40.47 | 0 | W | 2 | 1 | 2 | 4 | NIL | NIL  (27.1)^i^ | No ethnic breakdown |
| 24 | Fraser & Pakenham | 2008 | 44 | 13 | 61 | W | 1 | NIL | NIL | 4 | 8 | 88  (70)^i^ | No ethnic breakdown |
| 25 | Fraser & Pakenham | 2009 | 44 | 13 | 61.4 | W | 1 | NIL | NIL | NIL | NIL | 88  (70)^i^ | No ethnic breakdown |
| 26 | Goetz et al. | 2020 | 68 | 32.07 | 100 | W | 2 | 1 | 2 | 1 | NIL | NIL  (57)^k^ | No ethnic breakdown |
| 27 | Graham et al. | 2020 | 146 | 43.3 | 81.5 | W | 2 | 1 | 2 | 8 | 16 | 92  (96)^j^ | 3%American Indian  1%Asian  32% Black/African American  65%White  4%Other/NA  7%Hispanic |
| 28 | Harrer et al. | 2021 | 200 | 36.97 | 85 | W | 2 | 2 | 3 | 7 | 3 | 83  (66)^j^ | No ethnic breakdown |
| 29 | Hay et al. | 2007 | 122 | 28.5 | 100 | W | 1 | NIL | NIL | 12 | 12 | 83.6  (83.6)^j^ | No ethnic breakdown |
| 30 | Hui et al. | 2015 | 197 | NIL | 64.96 | E | 2 | 2 | 1 | 6 | NIL | 62.6  (55.1)^j^ | No ethnic breakdown |
| 31 | Hung et al. | 2021 | 358 | 20.8 | 82.4 | E | 1 | NIL | NIL | 4 | 4 | 93.8  (91.8)^j^ | No ethnic breakdown |
| 32 | Hurley et al. | 2021 | 540 | 47.7 | 59.4 | W | 1 | NIL | NIL | 1 | NIL | 30.9  (64.2)^i^ | No ethnic breakdown |
| 33 | Immura et al. | 2016 | 1236 | 39.5 | 29.6 | E | 2 | 2 | 2 | 16 | 16 | 87.2  (77.8)^j^ | No ethnic breakdown |
| 34 | Johansson et al. | 2022 | 144 | 63 | 38 | W | 2 | 2 | 4 | 9 | NIL | NIL  (NIL)^j^ | No ethnic breakdown |
| 35 | Jones et al. | 2020 | 57 | 16.3 | 79 | W | 2 | 1 | 2 | 8 | NIL | NIL  (79.5)^j^ | 95% white;  5% others |
| 36 | Kahlke et al. | 2019 | 200 | 26.7 | 62 | W | 1 | NIL | NIL | 10 | NIL | 94  (91)^j^ | No ethnic breakdown |
| 37 | Kawadler et al. | 2020 | 55 | 34.67 | 54.55 | W | 2 | 1 | 2 | 4 | NIL | NIL  (84.6)^j^ | No ethnic breakdown |
| 38 | Kitchener & Jorm | 2004 | 301 | NIL | 78.1 | W | 1 | NIL | NIL | 3 | NIL | 100  (100)^j^ | No ethnic breakdown |
| 39 | Klim-Conforti et al. | 2021 | 430 | NIL | 61.8 | W | 1 | NIL | NIL | 12 | NIL | 84.2  (60.6)^i^ | No ethnic breakdown |
| 40 | Koike et al. | 2018 | 259 | 20 | 42.1 | E | 2 | 2 | 3 | 48 | NIL | 83.9  (83.7)^i^ | No ethnic breakdown |
| 41 | Kuhn et al. | 2017 | 120 | 39 | 69.2 | W | 2 | 2 | 1 | 12 | 24 | 87.9  (75.8)^j^ | 66.1%White  14.5% African American or Black  11.3% Asian  21% Hispanic  3.2% American Indian or Alaskan Native  0% Asian Indian  3.2% Pacific Islander |
| 42 | Lai et al. | 2022 | 48 | 20.47 | 77.08 | E | 1 | NIL | NIL | 18 | NIL | NIL  (80)^j^ | No ethnic breakdown |
| 43 | Lattie et al. | 2020 | 20 | 24.19 | 85 | W | 2 | 1 | 2 | 8 | 4 | NIL  (95)^j^ | No ethnic breakdown |
| 44 | Levin et al. | 2016 | 234 | 21.61 | 76.9 | W | 2 | 3 | 3 | 3 | 4,12 | 63  (72)^i^ | 76.2% White or Caucasian,  9.3% Asian,  3.5% Black or African American, 1.8% American Indian/Alaska Native,  1.3% Native Hawaiian or Other Pacific Islander,  7.9% other racial background,  16.2% Hispanic or Latino |
| 45 | Lillevoll et al. | 2014 | 1337 | 16.8 | 44.12 | W | 2 | 1 | 2 | 6 | NIL | 74.4  (70)^j^ | No ethnic breakdown |
| 46 | Lindow et al. | 2020 | 436 | 14.5 | 61.3 | W | 1 | NIL | NIL | 5 | NIL | NIL  (84)^j^ | No ethnic breakdown |
| 47 | Lintvedt et al. | 2013 | 163 | 28.2 | 76.7 | W | 2 | 2 | 1 | 8 | NIL | 100  (100) | No ethnic breakdown |
| 48 | Loreto | 2018 | 94 | 19 | 53.2 | W | 1 | NIL | NIL | 1 | NIL | 95.8  (67.4) | No ethnic breakdown |
| 49 | MacLean et al. | 2020 | 95 | 44.2 | 70 | W | 3 | 2 | 4 | 12 | NIL | 83.3  (91.5)^j^ | 1%First Nations  0% Inuk  3%Métis  3% Asian  0%African, Caribbean, or Black  87%White  5% Other |
| 50 | Mailey et al. | 2019 | 231 | 32.5 | 100 | W | 2 | 2 | 3 | 10 | NIL | 67  (75.5)^k^ | 91%White |
| 51 | Mak et al. | 2018 | 2282 | 31.9 | 54.6 | E | 2 | 3 | 4 | 4 | 4,16 | 17  (13.1)^k^ | No ethnic breakdown |
| 52 | Mehrotra et al. | 2018 | 78 | 32.28 | 48.7 | E | 2 | 1 | 2 | 8 | 8,16 | NIL  (75)^i^ | No ethnic breakdown |
| 53 | Milgrom et al. | 2020 | 43 | 31.6 | 100 | W | 2 | 2 | 4 | 12 | 12 | 100  (90.48)^j^ | No ethnic breakdown |
| 54 | Moeini et al. | 2019 | 128 | 16.2 | 100 | E | 2 | 2 | 2 | 24 | NIL | 78.1  (81)^j^ | No ethnic breakdown |
| 55 | Moessner et al. | 2016 | 453 | 15.7 | 72.14 | W | 2 | 2 | 2 | 12 | NIL | 100  (100) | No ethnic breakdown |
| 56 | Moser et al. | 2019 | 98 | 40.4 | 85.7 | W | 2 | 3 | 4 | 4 | 12 | 70  (25)^i^ | No ethnic breakdown |
| 57 | Nguyen-Feng et al. | 2017 | 365 | NIL | 75 | W | 2 | 2 | 3 | 4 | 3,5 | 61.20  (73)^i^ | 73% White  17% Asian  3% Hispanic  7% other categories |
| 58 | O'Dea et al. | 2020 | 193 | 14.32 | 86.53 | W | 2 | 1 | 2 | 4 | NIL | 73.2  (68.7)^i^ | No ethnic breakdown |
| 59 | O'Dea et al. | 2021 | 1802 | 14.3 | 51.6 | W | 2 | 2 | 2 | 12 | NIL | 71.5  (44)^i^ | No ethnic breakdown |
| 60 | Pearce et al. | 2003 | 42 | 24.2 | 71.4 | W | 1 | NIL | NIL | 2 | NIL | NIL  (100) | No ethnic breakdown |
| 61 | Perry et al. | 2014 | 380 | 14.75 | 50 | W | 1 | NIL | NIL | 8 | 24 | 40.5  (66.7)^k^ | No ethnic breakdown |
| 62 | Perry et al. | 2018 | 34 | NIL | 55.88 | W | 1 | NIL | NIL | 8 | NIL | NIL  (94.12)^j^ | No ethnic breakdown |
| 63 | Persson Asplund et al. | 2018 | 117 | 46.9 | 67 | W | 3 | 1 | 3 | 8 | 32 | 49.4  (64.4)^i^ | No ethnic breakdown |
| 64 | Radovic et al. | 2021 | 38 | 15.9 | 76 | W | 2 | 2 | 4 | 12 | 6 | 55  (77.8)^k^ | 72%White  6% Black or African American  22% >1 race  0% Asian, American Indian or Alaska native |
| 65 | Reupert et al. | 2020 | 31 | 21.83 | 96.77 | W | 2 | 1 | 2 | 6 | 2 | NIL  (60.8)^j^ | 58.1%Australian  16.1%White European  9.7%Asian  3.2% Indian  3.2% Middle Eastern  3.2% American  3.2% Other  3.2% Not specified |
| 66 | Rodante et al. | 2020 | 18 | 31.82 | 80.95 | W | 3 | 2 | 3 | 4 | NIL | 95.5  (26.5)^j^ | No ethnic breakdown |
| 67 | Shehadeh et al. | 2019 | 126 | 27.7 | 78 | E | 2 | 1 | 2 | 8 | NIL | NIL  (50)^k^ | 88% Lebanese  6%Palestinian  4% Syrian  2% Other |
| 68 | Sin et al. | 2022 | 407 | 53.14 | 81.03 | W | 2 | 2 | 3 | 40 | 40 | 73.4  (85.3)^i^ | 87% White;  4% mixed;  10%Asian  8% Back;  1% others |
| 69 | Tay et al. | 2022 | 174 | NIL | 71.26 | E | 2 | 2 | 2 | 2 | 6 | 100  (100) | Chinese 90.63%  Malay 2.08%  Indian 6.25%  Others 1.04% |
| 70 | Tighe et al. | 2017 | 61 | 26.25 | 63.93 | W | 2 | 1,2 | 1,1 | 6 | NIL | 100  (93.5)^j^ | 90%Aboriginal  2% Torres Strait Islander  2% Both aboriginal and Torres Strait Islander  6.6% Neither aboriginal or Torres Strait Islander |
| 71 | Toland | 2011 | 87 | NIL | 4.6 | W | 1 | NIL | NIL | NIL | NIL | NIL  (43.9)^j^ | No ethnic breakdown |
| 72 | Van Voorhees et al. | 2012 | 50 | 29.57 | 10.2 | W | 2 | 1 | 1 | 12 | NIL | NIL  (58)^i^ | 6.12% African American  8.16% Asian  10.20% Hispanic  73.47% White  2.04% Other |
| 73 | Vechiu | 2021 | 110 | 46.49 | 76 | W | 2 | 2 | 1 | 1 | NIL | 93.4  (91.4)^i^ | 64%White/ Caucasian  12%Hispanic/Latino  12%Black/African-American  5%Asian/Asian-American  7% Other |
| 74 | Walker et al. | 2010 | 900 | 65.9 | 60.2 | W | 1 | NIL | NIL | 6 | 12,24 | 91.5  (86.5)^i^ | No ethnic breakdown |
| 75 | Wang & Du | 2020 | 374 | 17.97 | 61.26 | E | 1 | NIL | NIL | 8 | NIL | 86.5  (100) | No ethnic breakdown |
| 76 | Woods et al. | 2020 | 75 | 37.3 | 0 | W | 1 | NIL | NIL | 40 | NIL | 100  (0) | No ethnic breakdown |
| 77 | Yamaguchi et al. | 2019 | 259 | 20 | 47.9 | E | 2 | 2,3 | 3,3 | 10 | 12,24 | 80.5  (71.1)^i^ | No ethnic breakdown |
| 78 | Zwerenz et al. | 2019 | 229 | 48 | 60.7 | W | 2 | 3 | 3 | 12 | 24 | 64  (75.6)^i^ | No ethnic breakdown |
| 79 | zhuang et al. | 2020 | 202 | 24.32 | 80.40 | E | 1 | 1 | NIL | 2 | 12 | 100  (51)^i^ | Mainland China (87.3%)  Hong Kong (3.9%)  Others (5.0%)  Missing (3.9%) |
| 80 | Hart et al | 2022 | 1942 | 15.82 | 43.94 | W | 1 | 2 | NIL | 14hours | 48 | 53  (57)^j^ | No ethnic breakdown |
| 81 | Li et al. | 2019 | 293 | 37.99 | 50.40 | E | 1 | 2 | NIL | 3hours | NIL | 78.2  (74.3)^j^ | No ethnic breakdown |
| 82 | Dimoff & Kelloway | 2019 | 100 | 42.58 | 33.30 | W | 1 | 1 | NIL | 3hours | 12 | (82)^j^ | No ethnic breakdown |
| 83 | Halsall et al | 2019 | 110 | 13-25 | 79.20 | W | 2 | 1 | 2 | 20 | NIL | (100) | No ethnic breakdown |
| 84 | Pottinger et al. | 2021 | 204 | 40-50 | NIL | W | 1 | 1 | NIL | 3days | NIL | (77)^j^ | No ethnic breakdown |
| 85 | Dimoff & Kelloway study 1 | 2016 | 70 | 49.28 | 45.80 | W | 1 | 1 | NIL | 3hours | 8 | (72.4)^j^ | No ethnic breakdown |
| 86 | Brooks et al. | 2023 | 78 | 14.00 | 48.70 | E | 2 | 1 | NIL | 4 | NIL | (89.7)^j^ | No ethnic breakdown |
| 87 | Skre et al. | 2013 | 1072 | 14.00 | 46.10 | W | 1 | 2 | 2 | 3days | 8 | 80.5 (76.7)^k^ | No ethnic breakdown |
| 88 | Li et al | 2014 | 99 | 34.80 | 48.50 | E | 1 | 1 | NIL | 1day | NIL | (100) | No ethnic breakdown |
| 89 | Morgaine et al | 2017 | 430 | NIL | NIL | W | 1 | 1 | NIL | 8 | NIL | (100) | No ethnic breakdown |
| 90 | Vella et al. | 2020 | 816 | 14.53 | NIL | W | 3 | 1 | 1 | 8 | NIL | 30 (30)^k^ | No ethnic breakdown |
| 91 | Yamaguchi et al | 2020 | 975 | NIL | NIL | E | 1 | 2 | NIL | 50min | 8 | 89 (90.6)^i^ | All Japanese |
| 92 | Higgins & Sharek | 2016 | 28 | 19-24 | NIL | W | 1 | 1 | NIL | 30hours | NIL | (93)^j^ | No ethnic breakdown |
| 93 | Choi | 2017 | 64 | 30-40 | 100 | E | 1 | 2 | NIL | 8 | NIL | 100 (99)^j^ | No ethnic breakdown |
| 94 | Yang et al. | 2018 | 48 | NIL | 89.60 | E | 1 | 1 | NIL | 3days | NIL | (77)^j^ | No ethnic breakdown |
| 95 | Chen et al. | 2023 | 190 | 36.50 | 68.65 | E | 2 | 1 | 1 | NIL | NIL | (100) | No ethnic breakdown |
| 96 | Jones et al | 2022 | 207 | 28.84 | 61.80 | W | 2 | 1 | 1 | NIL | NIL | (53.7)^j^ | No ethnic breakdown |
| 97 | Rosen et al | 2022 | 173 | NIL | NIL | W | 3 | 3 | 4 | 540mins | NIL | 21.8 (65.8)^j^ | No ethnic breakdown |
| 98 | Tay et al. | 2022 | 179 | 18-24 | 62.30 | E | 2 | 2 | 4 | 2 | 8 | 100 (99)^i^ | Chinese (90.63%) Malay (2.08%) Indian (6.25%) Others (1.04%) |
| 99 | Li et al | 2013 | 127 | 20.82 | 57.50 | E | 2 | 1 | 2 | 3 | NIL | (57.5)^k^ | No ethnic breakdown |
| 100 | Curran | 2023 | 71 | 19.20 | 71.80 | W | 2 | 1 | 1 | 30mins | NIL | (100) | White: English/Welsh/Scottish/Northern 49.3% Irish/British Any other White background 2.8% Mixed/Multiple ethnic groups: White and Black Caribbean 1.4% White and Black African 4.2% White and Asian 1.4% Asian Indian 8.5% Pakistani 4.2% Bangladeshi 1.4% Chinese 1.4% Any other Asian background 4.2% Black/African/Caribbean/Black British: African 2.8% Caribbean 1.4% Any other Black/African/ Caribbean background 1.4% Other ethnic group: Arab 2.8% Prefer not to say 4.2% |
| 101 | Armstrong et al. | 2011 | 70 | 37.00 | 86.40 | E | 2 | 1 | NIL | 4days | 12 | (94.3)^j^ | No ethnic breakdown |
| 102 | Milin et al | 2016 | 534 | less than 18 | 55.10 | W | 3 | 1 | 1 | 8 | NIL | (87.8)^j^ | No ethnic breakdown |
| 103 | Gilam et al. | 2018 | 251 | NIL | 74.50 | W | 1 | 1 | NIL | less than 1 week | NIL | (100) | No ethnic breakdown |
| 104 | Hunt et al | 2019 | 71 | NIL | NIL | W | 1 | 1 | NIL | 16 | NIL | (100) | No ethnic breakdown |
| 105 | Kutcher | 2015 | 175 | NIL | NIL | W | 1 | 1 | NIL | 8 | 8 | (65)^j^ | No ethnic breakdown |
| 106 | Patalay | 2017 | 50 | 20.97 | 65.00 | W | 1 | 1 | NIL | 3 | NIL | (60)^i^ | 46.5% of school students identified as White, 16.1% as Black and 14.8% as Mixed. Others identified as Asian (10.9%) and or other ethnicities (11.7%) |
| 107 | Mutiso et al | 2018 | 5728 | 20-60 | 65.10 | South Africa | 1 | 1 | NIL | 45mins | 12 | (71.4)^k^ | No ethnic breakdown |
| 108 | Queroue et al | 2021 | 101 | 21.00 | 78.00 | W | 2 | 1 | 1 | 30mins | NIL | (100) | No ethnic breakdown |
| 109 | Arthur et al | 2022 | 149 | 18-50 | 12.90 | W | 2 | 1 | 2 | 3hours | 12 | (90)^K^ | No ethnic breakdown |
| 110 | Lexen et al. | 2021 | 94 | 42.00 | 70.00 | W | 1 | 1 | NIL | 17hours | 24 | (100) | No ethnic breakdown |
| 111 | Amaral et al | 2020 | 14 | 51.00 | 84.60 | W | 1 | 1 | NIL | 26hours | NIL | (100) | No ethnic breakdown |
| 112 | Zare et al. | 2021 | 220 | 13-15 | 100.00 | E | 1 | 1 | NIL | 90mins | NIL | (100) | No ethnic breakdown |
| 113 | Liu | 2021 | 305 | 20-40 | 88.30 | W | 2 | 2 | 2 | 30mins | NIL | 46 (69)^j^ | No ethnic breakdown |
| 114 | Liddle et al. | 2021 | 102 | 14.30 | 0.00 | W | 1 | 1 | NIL | 45mins | 4 | 63.5 (72.9)^j^ | No ethnic breakdown |
| 115 | Gratwick-Sarll and Bentley | 2014 | 177 | 20.30 | 95.90 | E | 1 | 1 | NIL | 3hours | 12 | (91.6)^j^ | No ethnic breakdown |
| 116 | Ayano et al. | 2017 | 94 | 27.80 | 70.20 | Ethopia | 1 | 1 | 2 | 5days | NIL | (100) | Amhara 25.53% Oromo 44.68% Tigray 15.96% Gurage 8.52% Others 5.31% |
| 117 | Bonnie et al. | 2019 | 45 | 25.80 | 60.00 | E | 2 | 1 | 1 | 1 | NIL | (100) | Asian (31%) Middle Eastern (8%) African (12%) European (15%) Indian (12%) South American (8%) Canadian (4%) Multiracial (8%) |
| 118 | Slewa-Younan | 202 | 54 | 47.06 | 69.00 | E | 1 | 1 | NIL | 1day | 24 | (96.3)^j^ | Iraq 35% Australia 25% Lebanon 15% |
| 119 | Reavley et al. | 2014 | 767 | 24.89 | 69.30 | W | 2 | 1 | 3 | 96 | 24 | (49.8)^k^ | No ethnic breakdown |
| 120 | Fung et al. | 2016 | 247 | 18-50 | 41.00 | E | 1 | 2 | NIL | 16.5hrs | NIL | 100 | No ethnic breakdown |
| 121 | Bella-Awusah | 2014 | 154 | 15.30 | 51.90 | South Africa | 1 | 1 | NIL | 3hours | 24 | (89.7)^j^ | No ethnic breakdown |
| 122 | Edgar et al. | 2021 | 875 | NIL | 71.40 | W | 1 | 2 | NIL | 48 | NIL | (83.3)^j^ | No ethnic breakdown |
| 123 | Mohatt et al | 2017 | 176 | NIL | NIL | W | 1 | 1 | NIL | 8hours | 32 | (68)^j^ | No ethnic breakdown |
| 124 | Farooq et al. | 2021 | 246 | NIL | NIL | E | 1 | 1 | NIL | 4hours | 24 | (56.1)^k^ | No ethnic breakdown |
| 125 | o'Reilly et al. | 2011 | 272 | 21.00 | 76.30 | E | 2 | 1 | 1 | 12hours | NIL | (86.4)^j^ | No ethnic breakdown |
| 126 | Gurung et al | 2020 | 458 | 32.20 | 47.80 | E | 1 | 1 | NIL | 40mins | NIL | (88.2)^i^ | No ethnic breakdown |
| 137 | Jorm et al | 2010 | 262 | 40.00 | 81.00 | E | 3 | 2,3 | 2,4 | 4 | 24 | 100 (100) | 91% were Australian citizens and 88% had English as their first language. |
| 128 | Lam et al. | 2010 | 108 | 15-55 | 79.70 | E | 1 | 1 | NIL | 8 | NIL | (100) | Mainland China 29.6% Hong Kong 44.4% Vietnam 9.3% Singapore 4.6% Taiwan 2.8% Other 7.4% Unknown 1.9% |
| 129 | Jordans et al | 2012 | 109 | 25-40 | 35.80 | E | 1 | 1 | NIL | 2days | 8 | (91.7)^j^ | No ethnic breakdown |
| 130 | Guajardo et al | 2019 | 372 | 14-17 | 50.50 | E | 1 | 3 | NIL | 14hours | 12 | 58.8 (68)^j^ | No ethnic breakdown |
| 131 | Patafio et al | 2021 | 330 | 13.73 | 42.00 | E | 1 | 2 | NIL | 8 | 16 | 45 (50)k | No ethnic breakdown |
| 132 | Tuijunam et al | 2022 | 185 | 13.43 | 45.40 | W | 2 | 2 | 2 | 1wk | 24 | 60 (67.3)^j^ | No ethnic breakdown |
| 133 | Tay et al. | 2019 | 68 | 30.26 | 63.20 | E | 1 | 1 | NIL | 8hr | NIL | (100) | No ethnic breakdown |
| 134 | Zamorski et al. | 2012 | 22113 | 26-37 or more | 8.20 | W | 3 | 3 | 1 | 2days | NIL | 75 (98)^j^ | No ethnic breakdown |
| 135 | oduguwa et al | 2017 | 205 | 45216 | 47.00 | South Africa | 1 | 1 | NIL | 5hours | 3 | (67.5)^j^ | No ethnic breakdown |
| 136 | Pinto-Foltz et al | 2011 | 156 | 15 | 69.00 | W | 1 | 1 | NIL | 60mins | 8 | (92)i | 69% white females |
| 137 | Bjørnsen et al | 2018 | 357 | 15-21 | 53.00 | W | 1 | 1 | NIL | 20 | NIL | (31)^j^ | No ethnic breakdown |
| 138 | Wilcox et al | 2023 | 1915 | 10-12th graders | 55.30 | W | 1 | 2 | NIL | 8hours | NIL | 27 out of 44 schools | White (47.0%) Black (6.8%) Hispanic (31.8%) Asian (8.8%) Native American (1.5%) Multiracial (4.2%) |
| 139 | Crooks et al | 2018 | 149 | 42.10 | NIL | W | 1 | 1 | NIL | NA | NIL | (96.7)^j^ | Indigenous background (81.3% versus 15.4%), with three participants (3.3%) not answering the question |
| 140 | Loureiro | 2020 | 219 | 17.99 | 84.50 | W | 1 | 1 | NIL | 9hours | NIL | (100) | No ethnic breakdown |
| 141 | Payne et al | 2001 | 954 | 40 | 92.60 | W | 1 | 1 | NIL | NIL | NIL | (55)^i^ | No ethnic breakdown |
| 142 | Gargia-Ortega et al | 2013 | 42 | NIL | NIL | W | 1 | 1 | NIL | 32 | NIL | (64.3)^j^ | No ethnic breakdown |
| 143 | DeSocio et al. | 2006 | 370 | 45211 | NIL | W | 1 | 1 | NIL | 6 | NIL | (100) | No ethnic breakdown |
| 144 | Repp et al. | 2019 | 488 | 40.30 | 61.08 | W | 1 | 1 | NIL | 8hours | 24 | (17)^j^ | Black/African American (56.08%) White (31.76%) Other (12.16%) Latinx (21.60%) |

^a^sample size

^b^percentage of adolescent sample who are female

^c^Culture = sample’s cultural background (E = Eastern; W = Western)

^d^Mode = mode of digital literacy accessed (1 = in-person; 2 = online; 3 = both in-person and online)

^e^intervention condition (1 = pre—post DMHL/traditional MHL; 2 = DMHL/traditional MHL vs. waitlist control; 3 = DMHL PLUS vs. non-DMHL (there are not studies that compared DMHL ONLY vs. non-DMHL)

^f^DMHL component (1=DMHL ONLY vs. waitlist control; 2=DMHL PLUS vs. waitlist control; 3=DMHL ONLY vs. DMHL PLUS; 4=DMHL PLUS vs. Non-DMHL)

^g^Duration = duration of intervention (in weeks, unless stated otherwise)

^h^Follow-up = follow-up measurements since the first measurement (in weeks)

^i^Baseline sample and sample of completers were similar on baseline and demographic measures (i.e, sample of completers was representative of the populations being targeted)

^j^Study did not provide information comparing baseline sample vs. sample of completers for baseline and demographic measures

^k^Baseline sample and sample of completers differ in baseline and/or demographic measures (i.e., sample of completers was not representative of the populations being targeted).

Reviewers extracted and coded data on study design, sample characteristics (e.g., age, gender, sample size, country), and mental health or well-being outcomes (e.g., depression, anxiety, loneliness, internalizing and externalizing symptoms, resilience, life satisfaction, and quality of life). Reviewers also coded theoretical/conceptual frameworks, conceptualizations and operationalizations of DMHL, DMHL components (i.e., DMHL ONLY vs. control, DMHL PLUS vs. control, DMHL ONLY vs. DMHL PLUS, DMHL ONLY vs. non-DMHL, and DMHL PLUS vs. non-DMHL), study designs (i.e., pre-post DMHL interventions, waitlist control vs. DMHL interventions, and DMHL vs. non-DMHL interventions), features and affordances of DMHL interventions (i.e., new platforms that afford greater interactivity, including mobile apps, web-based/internet platforms, and social media vs. conventional platforms that afford lower interactivity, including films, videos, multimedia, and emails), dosage and carry-over effects of DMHL interventions. Reviewers extracted and computed Cohen’s D (to address H1, RQ1b, RQ1c) and odd-ratios (to address RQ1a on the effect of DMHL interventions on uptake) from DMHL interventions.

The checklist utilizes 27 items and assessed studies across five domains of quality: external validity, study bias, confounding, selection bias, and study power. Twenty-six items on this checklist requires yes (1) or no (0) answers in response to questions regarding study quality (e.g., “Is the hypothesis/aim/objective of the study clearly described?”). An additional item was rated on a 3-point scale (yes=2, partial=1, and no=0), generating scores range from 0 to 28 with higher scores indicating greater quality. Based on the quality assessments, most studies indicated moderate to high quality (M = 16.91, SD = 4.15) and had scores that ranged from 10 to 23. We further assessed DMHL interventions using the Cochrane Collaboration’s risk of bias tool [80], with five domains of bias: (1) random sequence generation, and allocation concealment, (2) blinding of participants and personnel, (3) blinding of outcome assessment, (4) incomplete outcome data, and (5) selective reporting and other bias. Of the 144 studies, only 21 (14.5%) had low risk of bias ratings across the five categories. 89 of the studies (61.8%) used random sequence generation, 21 (14.6%) had biases in allocation concealment, 40 (27.8%) had biases in blinding of participants and personnel, 55 (38.2%) had biases in blinding of outcome assessment, 98 (68.2%) had incomplete outcome data, 2 (2.5%) had selective reporting and other bias. Only 52 (36.1%) blinded both participants and personnel to the condition allocation (performance bias), 31 (21.5%) blinded outcome assessment, 67 (46.5%) demonstrated low reporting bias by preregistering or making their study protocol available and by reporting all the primary outcomes. About one third of all ratings (38.3%, 220 out of 575; 575 = total number of ratings conducted) were unclear or characterized as having high risk of bias.

47 studies examined traditional, face-to-face mental health literacy and produced 102 effect sizes, with a total of 27,692 participants [e.g., 10,87]. 59 studies that examined DMHL provided 87 effect sizes, with a total of 21,213 participants (e.g., [20,88]), and 8 studies that examined both digital and traditional mental health literacy produced 17 effect sizes, with a total of 24,128 participants (e.g., [89,90]). These data were obtained from studies conducted across 27 countries (Argentina, Portugal, Ethiopia, Nigeria, Australia, Austria, Canada, China, Germany, Hong Kong, India, Indonesia, Iran, Ireland, Japan, Lebanon, Palestine, Norway, Poland, Singapore, Sweden, Switzerland, Syria, Taiwan, United Kingdom, United States). The majority of included DMHL studies were from Western cultural contexts (74%; n=56), with more than half of the study participants from the United Kingdom (55.3%; n=42) or the United States of America, with 3 studies from South America—Argentina and Portugal (3.9%). Approximately a third of the studies were from Asian cultural contexts (22.4%; n=17) and Australia (17.1%; n=13), and only 4 studies from countries in Africa (5.3%). Out of the 76 DMHL studies, 36 provided information on ethnic/racial make-up of the samples. 20 studies were conducted in Western cultural contexts, with samples that were predominantly White (ranged from 54.5% to 95%) and the rest comprised of African American (ranged from 0.8% to 32% ), Asian American (ranged from 1% to 51%), and Hispanic (0.5% to 56.8%), and the remaining studies were from Indonesia, Australia, Lebanon, Singapore, Ethiopia (refer to Multimedia Appendix 2 for ethnic composition of these studies), The average age of the sample for the examined studies ranged from 12.21 to 65.9. Gender was only reported as binary with the female proportion ranging from 0% to 100%.

For DMHL interventions, uptake ranged from 13.1% to 100%, nineteen interventions produced 31 effect sizes for both proximal and distal outcomes, and 41 produced 48 effect sizes for distal outcomes only. For traditional, face-to-face MHL interventions, 22 produced 31 effect sizes for both types of outcomes, and 13 provided 17 effect sizes for distal outcomes only. Our study included 17 DMHL ONLY interventions (vs. control) that produced 31 effect sizes, 29 interventions with DMHL PLUS (vs. control) that produced 35 effect sizes, 14 interventions with DMHL ONLY vs. DMHL PLUS that provided 19 effect sizes, and no interventions with DMHL ONLY vs. non-DMHL, and 13 interventions with DMHL PLUS vs. non-DMHL that produced 17 effect sizes. 27 DMHL interventions included in this review produced 34 effect sizes that involved pre vs. post intervention comparison (e.g. [48, 91]), and 28 interventions produced 43 effect sizes that involved control vs. intervention groups (e.g., [16,54]).

For DMHL interventions, 44 involved new platforms that afford greater interactivity (n=15 mobile apps, n=35 web-based/internet platforms, and social media, and n=2 a combination of mobile apps, web-based/internet platforms, and social media) and 13 used conventional platforms that afford lower interactivity (i.e., films, videos, multimedia, and emails). Overall, the studies allocated21,213 participants to DMHL interventions and 34,349 participants to waitlist controls. The mean duration of DMHL interventions was 10 weeks (SD=11.46; range=1 to 96 weeks), and that of traditional MHL interventions was 8 weeks (SD=15.12; range=1 to 96 weeks). 64 DMHL interventions measured post-intervention effects and 29 studies measured follow-up effects, and 70 traditional face-to-face MHL interventions assessed post intervention effects and 24 interventions assessed follow-up effects. The mean follow-up assessment for DMHL interventions was 18.1 weeks and the range was 4 to 34 weeks, and for traditional MHL interventions, mean follow up assessment was 20.6 weeks, with a range of 4 to 96 weeks.

Multimedia Appendix 2.

Table S2. Main frameworks of included studies

| **No.** | **Author(s)** | **Year** | **Theory/framework** | **Details of theory/framework** |
| --- | --- | --- | --- | --- |
| 1 | Ali et al. | 2014 | Person-centred web-based support | • Person-centred web-based support offers strategies for finding social support, to reduce time spent searching for informational and support resources online. |
| 2 | Alvarez-Jimenez et al. | 2021 | Moderated online social therapy | • Horyzons is based on the moderated online social therapy (MOST) model, which integrates interactive online therapy (“pathways” and “steps”), peer-to-peer online social networking (“the café”), peer moderation, and expert support. |
| 3 | Andrews et al. | 2022 | School-based mental health interventions | • School-based mental health interventions can provide support to individuals who have weak social connections. |
| 4 | Arjadi et al. | 2018 | Digital technology for treatment and prevention of mental disorders | • The main evidence base for the study was based on a systematic review published in 2017 regarding all forms of digital technology for treatment and prevention of mental disorders in low-income and middle-income countries. |
| 5 | Bakker et al. | 2018 | Self-guided interventions | • Self-guided interventions are part of a stepped-care approach, which prioritises "high and low intensity" psychological interventions, according to severity of mental health distresses and clinical needs. |
| 6 | Baumeister et al. | 2021 | Internet and mobile-based interventions | • Internet and mobile-based interventions have been suggested as promising approaches to close the gap in mental health provision due to its accessibility and efficacy. |
| 7 | Bjornsen et al. | 2018 | School-based mental health literacy programs | • School-based MHL programs are ones that promote mental health literacy and mental health promotion through the use of school activities and curriculum. |
| 8 | Blaney et al. | 2021 | Worry and Sadness Program | • The Worry and Sadness program is a non-therapist assisted internet Cognitive Behavioural Therapy intervention for anxiety. |
| 9 | Booth et al. | 2022 | Peer-led health education | • Peer-led health education is able to support in delivering sensitive information which may be more easily shared amongst peers the same age. |
| 10 | Carl et al. | 2020 | Cognitive behavioural therapy | • Cognitive behavioral therapy (CBT) is a psycho-social intervention that aims to reduce symptoms of various mental health conditions, primarily depression and anxiety disorders.  • CBT focuses on challenging and changing cognitive distortions (such as thoughts, beliefs, and attitudes) and their associated behaviors to improve emotional regulation and develop personal coping strategies that target solving current problems |
| 11 | Carr et al. | 2018 | Mental health literacy framework | • Mental health literacy has been defined as understanding how to obtain and maintain good mental health, understanding mental disorders and their treatments, developing capacities to decreasing stigma, and developing capacities to enhance help-seeking efficacy (knowing when, where, and how to seek help). |
| 12 | Castro et al. | 2012 | Strength-based approach | • Post-deployment resilience training uses a strength-based approach, and is designed to enhance soldier mental skill development, adaptation to the stressors of combat, and management of the transition from combat to home. |
| 13 | Cernvall et al. | 2018 | Internet psychological interventions | • The PTSD Coach app provides psychoeducational information about PTSD and strategies for coping with PTSD symptoms. |
| 14 | Chrisholm et al. | 2016 | Intergroup contact theory | • Intergroup contact theory posits that interaction between different groups reduces conflict, prejudice and discrimination. |
| 15 | Cieslak et al. | 2016 | Self-efficacy and social cognitive theory | • Self-efficacy refers to one's beliefs about their ability to cope with stressors and demands in life, in turn, enabling them to overcome stressors more effectively. stressors). Those beliefs enable individuals to deal more effectively with stressors (including traumatic events) and promote health and well-being. |
| 16 | Clough et al. | 2020 | Mental health literacy | • Jorm et al. (1997) posited this comprehension of mental health consists of several elements, including: (a) knowing how to identify symptoms of mental health-related problems and where to get information about them; (b) understanding the causes and risks associated with mental health-related problems; (c) knowledge and beliefs about self-help interventions versus professional care and its availability; and (d) knowledge and beliefs that influence help-seeking attitudes, intentions, and behaviors. |
| 17 | De Silva | 2022 | Social cognitive theory | • Social cognitive theory posits that one's knowledge is shaped by their environment (through social interactions, experiences and outside media influences). |
| 18 | DeLuca | 2021 | Contact intervention | • Using NAMI’s contact interventions as a framework (which were atheoretically designed), Pinto-Foltz and Logsdon (2009) proposed three model constructs to effectively reduce stigma—learning, persuasion, and stage of development—and concurrent empirical indicators to measure outcomes. |
| 19 | Ebert et al. | 2021 | Resilience model | • Resilience enables participants to cope with perceived stress by dealing with stressors without the help of additional psychological interventions. |
| 20 | Ebert et al. | 2016 | Lazarus and Folkman transactional model of stress | • The transactional model of stress and coping contends that a person's capacity to cope and adjust to challenges is a consequence of transactions (or interactions) that occur between a person and their environment. |
| 21 | Espie et al. | 2019 | Digital cognitive behavioural therapy | • Digital cognitive behavioural therapy are rendered through automated web platforms or a mobile app. It is used to improve night-time symptoms of insomnia. |
| 22 | Eustis et al. | 2018 | Acceptance-based behavioural web-based approach | • Acceptance-based approaches empowers clients to feel emotions and bodily sensations more experientially and without avoidance, and to be aware of the presence of thoughts without resistance and denial. |
| 23 | Fogarty et al. | 2017 | Web-based cognitive behavioral therapy programs | • Web-based CBT programs targeting depression and anxiety supports in reducing symptoms and improve work and social functioning. |
| 24 | Fraser & Pakenham | 2008 | Resilience framework | • Koping Adolescent Group Program (KAP) adheres to a resilience framework, and consequently is designed to improve adjustment outcomes for children of parents with a mental illness by modifying risk factors such as social isolation and inadequate mental health literacy and by strengthening protective factors such as an adequate repertoire of coping skills and intact peer relationships. |
| 25 | Fraser & Pakenham | 2009 | Resilience framework | • Koping Adolescent Group Program (KAP) adheres to a resilience framework, and consequently is designed to improve adjustment out- comes for children of parents with a mental illness by modifying risk factors such as social isolation and inadequate mental health literacy and by strengthening protective factors such as an adequate repertoire of coping skills and intact peer relationships. |
| 26 | Goetz et al. | 2020 | Mindfulness-based interventions | • Mindfulness-based interventions (MBIs) refer to interventions that touch on internal experiences - inclusive of cognitions, emotions, sensations, affect regulation, decision-making, self-management, and relaxation. |
| 27 | Graham et al. | 2020 | Digital mental health interventions | • Digital mental health interventions (DMHIs) are internet-based and mobile tools to improve mental health challenges. |
| 28 | Harrer et al. | 2021 | Internet-based stress management interventions | • Internet-based stress management interventions are interventions which convey techniques to cope with modifiable or non-modifiable stressors. |
| 29 | Hay et al. | 2007 | Mental Health Literacy | • Jorm (2000) has argued that one reason for unmet need for treatment in mental health is poor ‘mental health literacy’ (MHL), where MHL refers to the knowledge and beliefs about mental disorders which aid their recognition, management or prevention. |
| 30 | Hui et al. | 2015 | Theory of Planned Behaviour | • The Theory of Planned Behaviour maintains that behaviour is directly predicted by the formation of an intention to undertake the behaviour, along with perceptions of control over one's ability to undertake the behaviour. In turn, intentions are predicted by attitudes toward the behaviour, subjective norms regarding the behaviour, and perceptions of behavioural control. |
| 31 | Hung et al. | 2021 | Mental Health First Aid | • The MHFA programme is an effective standardised psychoeducational training programme that aims to enhance attendees’ knowledge of general mental health and common mental problems such as anxiety, depression, and psychosis, and the self-harming behaviours that can be triggered by these problems, with the aim of supporting people with mental health problems |
| 32 | Hurley et al. | 2021 | Mental health literacy framework | • The mental health framework (Jorm et al., 1997) was used to inform the intervention content, including improving knowledge, challenging misconceptions, and communicating about mental health to improve confidence and intentions to provide help. |
| 33 | Immura et al. | 2016 | Primary prevention intervention | • Primary prevention interventions can be classified into three categories: indicated, selective, and universal (Cuijpers et al., 2008; van Zoonen et al., 2014). Indicated prevention targets individuals who have some symptoms of a mental disorder but do not meet diagnostic criteria. Selective prevention focuses on individuals who are in a high risk group but have not yet developed a mental disorder. Universal prevention targets the general population, regardless of whether they have a higher risk of developing a disorder. |
| 34 | Johansson et al. | 2022 | Internet-based cognitive behavioral therapy | • Internet-based cognitive behavioral therapy (iCBT) through internet-based interventions, proven to be effective in reducing depressive symptoms and improving one's physical wellbeing. |
| 35 | Jones et al. | 2020 | Psyeducational interventions | • Psychoeducational interventions effective in enhancing mental health treatments and psychosocial functioning. |
| 36 | Kahlke et al. | 2019 | Internet- and mobile-based interventions | • Internet- and mobile-based interventions (IMIs) have proven effective in treatment of a range of mental health disorders. |
| 37 | Kawadler et al. | 2020 | Circumplex Model of Affect | • The circumplex model of affect proposes that all affective states arise from cognitive interpretations of core neural sensations that are the product of two independent neurophysiological systems.  • The app allows individuals to log their mood in the moment, and reflect back on their entries at a later date to gain insights into patterns and themes, and diaphragmatic breathing exercises for stress reduction. |
| 38 | Kitchener & Jorm | 2004 | Mental Health First Aid Training | • The course covers helping people in mental health crises and/or in the early stages of mental health problems. It trains participants on crisis situations, mental health problems, co-morbidity, along with the symptoms of these disorders, possible risk factors, where and how to get help and evidence-based effective help. |
| 39 | Klim-Conforti et al. | 2021 | School-based interventions (involving cognitive behavioural therapy) | • School-based interventions which involves the use of cognitive behavioural therapy help enhance existing suicide prevention efforts. |
| 40 | Koike et al. | 2018 | Filmed social contact interventions | • Filmed social contact (FSC) interventions using DVDs and/or websites effective in decreasing stigma and social withdrawals from persons with mental illness. |
| 41 | Kuhn et al. | 2017 | Smartphones as platforms for evidence-based PTSD treatments | • It has been found that smartphones have the potential to address mental health needs of those with PTSD, particularly with high demands from smartphone users to address mental health needs. |
| 42 | Lai et al. | 2022 | Mental health promotion and education programs | • Study assumes that the starting of mental health promotion and education programs at the university level can prevent public health students from being unable to complete their studies due to mental health struggles. |
| 43 | Lattie et al. | 2020 | Digital mental health programmes | • Digital mental health programs offer the potential to provide self-management tools and help triage students to appropriate services on campus, thus lessening the burden that counseling centers may face from students with low-level concerns and increasing the number of students who can receive support. |
| 44 | Levin et al. | 2016 | Acceptance and commitment therapy | • Acceptance and commitment therapy (ACT) (Hayes, Strosahl, & Wilson, 2012) seeks to reduce psychological inflexibility by increasing psychological flexibility, a therapeutic process of change whereby individuals learn to engage in valued patterns of action while being willing to experience whatever thoughts and feelings might arise. |
| 45 | Lillevoll et al. | 2014 | Internet-based cognitive behavioural therapy | • Internet-based cognitive behavioural therapy (ICBT) refers to the usage of internet for mental health interventions, and proven to be a promising approach to overcoming barriers to help-seeking among adolescents. |
| 46 | Lindow et al. | 2020 | Mental health literacy | • Jorm et al. (1997) posited this comprehension of mental health consists of several elements, including: (a) knowing how to identify symptoms of mental health-related problems and where to get information about them; (b) understanding the causes and risks associated with mental health-related problems; (c) knowledge and beliefs about self-help interventions versus professional care and its availability; and (d) knowledge and beliefs that influence help-seeking attitudes, intentions, and behaviors. |
| 47 | Lintvedt et al. | 2013 | Internet-based self-help intervention programmes | • Internet-based self-help intervention programmes typically support users in cognitive behavioural therapy (CBT) skills. |
| 48 | Loreto | 2018 | Health Belief Model | • Health Belief Model (Rosenstock, 1974) posits that one's beliefs regarding a health concern will directly influence capabilities to assess perceived benefits and susceptibility to an illness and predict proactive healthy behaviours. |
| 49 | MacLean et al. | 2020 | Web-based therapy guided model | • The delivery of web-based therapy as a treatment for depression can be performed in one of two ways: through the use of supports to assist patients through the web-based therapy (guided model) or through the self-help use of computerized treatment (unguided model). In the guided model, patients are provided support as they progress through web-based therapy. |
| 50 | Mailey et al. | 2019 | Self-Determination Theory | • Self-Determination Theory (SDT) enables autonomous motivation by fulfilling three core needs for optimal human functioning: autonomy, competence, relatedness |
| 51 | Mak et al. | 2018 | Mobile app-based mental health programs | • These programs are specifically targeted towards mindfulness, self-compassion and cognitive behavioural psychoeducation training to improve mental wellbeing of users. |
| 52 | Mehrotra et al. | 2018 | Internet-based self-help interventions | • Internet-based self-help intervention aids in addressing unmet mental health needs in low-resource settings with limited availability of mental health professionals and poor access to mental health services. |
| 53 | Milgrom et al. | 2020 | MumMoodBooster | • MumMoodBooster is a postnatal intervention with low-intensity guided support based on cognitive behavioral therapy (CBT). It was adapted from the Getting Ahead of Postnatal Depression program, which is specifically adapted for the needs of postnatal women (eg, presenting behavioral strategies before cognitive content. |
| 54 | Moeini et al. | 2019 | Social Cognitive Theory | • Social Cognitive Theory (SCT) posits that one's knowledge is shaped by their environment (through social interactions, experiences and outside media influences). |
| 55 | Moessner et al. | 2016 | Internet-based interventions | • Internet-based interventions support prevention, self-help, treatment, aftercare, and disease management. |
| 56 | Moser et al. | 2019 | Theoretical model of Adjustment Disorder | • The intervention is based on a manual by Bachem and Maercker. This manual is aimed at AjD for burglary victims and has already been successfully tested in a paper-based version. Based on the theoretical model of AjD for the ICD-11, it integrates evidence-based techniques from the areas of post-traumatic stress disorder, anxiety disorders and depression. |
| 57 | Nguyen-Feng et al. | 2017 | Temporal model of control over stressors | • The model maintains that control beliefs can focus on the past, present or future. Research testing this model has found that present control is the only form of control associated with less distress, that this relation remains after controlling for a range of other variables known to be related to distress (e.g., social support, coping, neuroticism), and that present control is one of the only factors associated with less distress (Frazier et al., 2011, 2012). |
| 58 | O'Dea et al. | 2020 | Social Learning Theory | • Social learning theory posits that learning takes place through observation, imitation and modeling. |
| 59 | O'Dea et al. | 2021 | Theory of help-seeking for mental health | • The service model is informed by Rickwood and colleagues (2005) theory of help-seeking for mental health, which characterises help-seeking as a translational process that involves awareness and appraisal of problems, expression of symptoms and need for support, availability of sources of help, and the willingness to disclose problems and seek out care. |
| 60 | Pearce et al. | 2003 | Theory of Planned Behaviour | • The Theory of Planned Behavior (Fishbein & Ajzen, 1975) maintains that behaviour is directly predicted by the formation of an intention to undertake the behaviour, along with perceptions of control over one's ability to undertake the behaviour. In turn, intentions are predicted by attitudes toward the behaviour, subjective norms regarding the behaviour, and perceptions of behavioural control. |
| 61 | Perry et al. | 2014 | Curriculum-based educational program | • Curriculum-based education serves enhance mental health knowledge, reduce stigma and improve access to care. |
| 62 | Perry et al. | 2018 | Strengths-based intervention with community-model | • As part of the community-model, study focuses on psychoeducation with primary aim and an emphasis on health, collaboration, acceptance and empowerment. Psychoeducational interventions consist of didactic skilful communication of key information and are based on the premise that the more knowledgeable and informed care recipients and caregivers are, the more positive, health-related outcomes will be. |
| 63 | Persson Asplund et al. | 2018 | Positive management tactics in occupational settings | • Positive management tactics in occupational settings has been linked to enhanced employee performance and mental wellbeing of employees. |
| 64 | Radovic et al. | 2021 | Supporting Our Valued Adolescents | • The Supporting Our Valued Adolescents (SOVA) intervention is a conceptual model that was designed to address these proposed targets by challenging health beliefs, promoting peer support, and encouraging parent-adolescent mental health discussion. |
| 65 | Reupert et al. | 2020 | Online intervention | • Online intervention targeting young adults who parents have a mental health struggle/substance can help support niche needs that they possess (e.g., mental health needs) and improve social connectedness/support. |
| 66 | Rodante et al. | 2020 | Mobile applications for evidence-based interventions | • Mobile applications have been applied in evidence-based interventions to be used for prevention of suicide behaviour |
| 67 | Shehadeh et al. | 2019 | Step-by-Step | • Step-by-Step is a brief, minimally guided self- help program for people with depression. is a five session online intervention using behavioral activation and stress management techniques designed to ameliorate symptoms of depression. |
| 68 | Sin et al. | 2022 | Psychoeducation interventions | • Psychoeducation interventions are able to provide information on psychosis and its management for carers. |
| 69 | Tay et al. | 2022 | Education programs disseminated via information and communication technology | • Education programs rendered through information and communication technology (ICT) can enhance mental health literacy. ICT could be referred to as telecommunication devices (e.g., robots, smartphones, computers, emails to deliver digital knowledge). |
| 70 | Tighe et al. | 2017 | Mobile and eHealth technologies | • Mobile and eHealth therapies can surmount confidentiality fears, while providing evidence-based therapeutic content, especially in areas where mental health services are scarce. |
| 71 | Toland | 2011 | Mental Health Literacy | • Jorm et al. (1997) posited this comprehension of mental health consists of several elements, including: (a) knowing how to identify symptoms of mental health-related problems and where to get information about them; (b) understanding the causes and risks associated with mental health-related problems; (c) knowledge and beliefs about self-help interventions versus professional care and its availability; and (d) knowledge and beliefs that influence help-seeking attitudes, intentions, and behaviors. |
| 72 | Van Voorhees et al. | 2012 | Theory of Planned Behavior | • The Theory of Planned Behavior assumes that individuals act rationally, according to their attitudes, subjective norms, and perceived behavioral control. These factors are not necessarily actively or consciously considered during decision-making, but form the backdrop for the decision-making process. |
| 73 | Vechiu | 2021 | Expanded Behavioral Model of Health Service Use Among Vulnerable Populations | • The Expanded Behavioral Model of Health Service Use Among Vulnerable Populations (EBMVP) is a conceptual framework for understanding health-seeking behaviors. It considers traditional and vulnerable predisposing, enabling, and need factors that may influence service users. |
| 74 | Walker et al. | 2010 | Social Cognitive Theory | • Social Cognitive Theory posits that learning occurs in a social context with a dynamic and reciprocal interaction of the person. |
| 75 | Wang & Du | 2020 | Mental health education | • Study identified pressing need to increase mental health literacy and enhance psychological health through mental health education in medical curriculum. |
| 76 | Woods et al. | 2020 | Sport-based interventions | • Sport-based interventions were chosen as sport may be more accepted by prisoners for mental health programmes. |
| 77 | Yamaguchi et al. | 2019 | Filmed social contact interventions | • In the filmed social contact group, participants watched a 30-min film which consisted of interviews of two men with schizophrenia, a portrayal of a woman with obsessive–compulsive disorder, and educational lecture slides with an audio explanation of mental health, accompanied by information on help-seeking and mental health service providers. |
| 78 | Zwerenz et al. | 2019 | Web-based self-help | • Deprexis is an interactive Web-based self-help program. It consists of 10 main modules plus an introductory and a summary module based on cognitive behavioral techniques, positive psychology, emotion-focused therapy, and dream work. |
| 79 | zhuang et al. | 2020 | Mental health first aid | Developed and implemented in Australia, MHFA) training has since been adopted in 21 other countries around the world. The effectiveness of MHFA training has been confirmed repeatedly through the culmination of empirical evidence since its establishment in 2001. |
| 80 | Hart et al | 2022 | Mental health first aid | MHFA is defined as the support provided to an individual who is developing a mental health problem, experiencing a worsening of an existing problem, or in a mental health crisis. |
| 81 | Li et al. | 2019 | NIL | NIL |
| 82 | Dimoff & Kelloway | 2019 | Resource utilization model | RUM is as a framework for better understanding how leaders can encourage employee resource use |
| 83 | Halsall et al | 2019 | Nudge theory and behavioral economics | Based on the premise that individuals often do not make rational choices. As such, it advocates for policy and practice that structures the environment to enforce automatic behaviors that enhance health. This strategy is in contrast to campaigns that seek to change behavior through increased knowledge and the enhancement of decision-making related to healthy behaviors. The concept of a nudge is defined as “any aspect of the choice architecture that alters people’s behavior in a predictable way without forbidding any options or significantly changing their economic incentive |
| 84 | Pottinger et al. | 2021 | Community health education | Community health education programmes can target the general public or can be location- or population-specific |
| 85 | Dimoff & Kelloway | 2016 | Self-efficacy | Self-efficacy applied to organizational leaders’ mental health literacy |
| 86 | Brooks et al. | 2023 | Mental health literacy framework | Mental health literacy framework in MLIC-low middle income country |
| 87 | Skre et al. | 2013 | Mental health literacy framework | The Norwegian health care system for mental health. n Norway, adolescents have easy access to the primary health care system through the school nurse, and all have a designated general practitioner |
| 88 | Li et al | 2014 | Guangzhou model | China also has initiated the “686” project to improve the community mental heath service. As one of the five national central cities, Guangzhou has developed a range of training courses and now is developing the “Guangzhou model” in the field of community mental health |
| 89 | Morgaine et al | 2017 | Mental health literacy framework | GoodYarn was initially developed by a Primary Health Organisation for a large farming region of southern New Zealand in response to the service gap of an accessible mental health literacy programme tailored to the specific needs of rural people |
| 90 | Vella et al. | 2020 | Mental health literacy framework | In sport, psychological resilience is defined as the mental processes (e.g., problem solving) and behaviors (e.g., accessing social support), which promote personal assets and protect an individual from the potential negative effect of stressors |
| 91 | Yamaguchi et al | 2020 | Mental health literacy framework | Embedding MHL educational programs delivered by school teachers within school curriculums |
| 92 | Higgins & Sharek | 2016 | Perinatal mental health problems | Delivery wives lack the knowledge and skills to be able to effectively and comprehensively assess and support women's mental health; research has also found that midwives lack awareness and confidence in referring women to appropriate resources |
| 93 | Choi | 2017 | Mental health literacy framework | Many immigrant women in international marriages experience difficulties due to limited health literacy |
| 94 | Yang et al. | 2018 | NIL | NIL |
| 95 | Chen et al., | 2023 | International mental health training center | The IMHTCT recruits trainee mental healthcare workers, including psychiatrists, nurses, clinical psychologists, social workers, and occupational therapists from Southeast Asian countries. The training programs provided by IMHTCT include clinical knowledge, practical skills, community-based practice, geriatric mental health, hospital management, and related knowledge. |
| 96 | Jones et al | 2022 | Rickwood’s Help-Seeking Model | This model describes that for individuals to seek help they must gradually transition from problem-solving at an intrapersonal level, to an interpersonal level. This transition involves the following four steps: developing awareness and appraisal of problems; the expression of symptoms and need for support; availability of sources of help; and a willingness to seek out and disclose to those sources. Additionally, early help-seeking can be improved by reducing negative beliefs associated with seeking psychological help, increasing social support, and improving mental health literacy |
| 97 | Rosen et al | 2022 | the certified nursing assistants (CNAs) education | To address the problem of providing consistent, high quality education to CNAs and other nursing personnel in nursing homes, we created a computer-based educational curriculum called “Solutions for Long-Term Care” (SLC). |
| 98 | Tay et al. | 2022 | NIL | NIL |
| 99 | Li et al | 2013 | Digital game-based learning. | benefits and educational effectiveness of digital game-based learning in both classroom and laboratory settings, and in various subjects like computer science, engineering, mathematics, and physics . The literature identifies motivational characteristics of educational computer games and also establishes characteristics that consistently facilitate game design and development |
| 100 | Curran | 2023 | NIL | NIL |
| 101 | Armstrong et al. | 2011 | Mental health literacy framework | mental health is to be successfully integrated in primary health care settings in low-income countries like India |
| 102 | Milin et al | 2016 | Mental health literacy framework | Mental health literacy encompasses knowledge and skills addressing biological and psychosocial aspects of mental health to improve understanding of mental health and mental disorders, to reduce stigma, to help with early identification of mental disorders, and to facilitate help seeking behaviors in youth. |
| 103 | Gilam et al. | 2018 | Mental health literacy framework | Transitions (2^nd^ edition) is a resource developed for use on college campuses respecting this MHL developmental focus. |
| 104 | Hunt et al | 2019 | Mental health literacy | As the foundation for mental health promotion, prevention and care, MHL has been recently defined as having four separate but related components: (1) obtaining and maintaining good mental health, (2) understanding mental disorders and their treatments, (3) decreasing stigma and (4) enhancing help-seeking efficacy. While increasing awareness of the need to address student MHL in post-secondary institutions is growing, there is a lack of evidence-based resources available to help address this need. |
| 105 | Kutcher | 2015 | NIL | NIL |
| 106 | Patalay | 2017 | School and university-based mental health education; | Many models of mental health literacy interventions that have been introduced in schools including ones that are teacher delivered (Milin et al., 2016), mental health professional, and other third party delivered (Chisholm et al., 2016; Pinfold et al., 2003). The empirical evidence for most school based mental health literacy interventions has been quite limited |
| 107 | Mutiso et al | 2018 | NIL | NIL |
| 108 | Queroue et al | 2021 | mental health literary framework | Previous studies have proved the efficacy of interventions targeting MHL among University students. These interventions were delivered face to face, online, or a combination, using different learning approaches including didactic sessions, interpersonal relation, cognitive-behavioral learning and gamification. In particular, two systematics reviews have established the effectiveness of web-based MHL interventions demonstrating that online programs and tools can address a large number of students without time and space constraints, preserve privacy and produce positive outcomes corresponding to enhanced knowledge, improve behavioral styles and decrease depressive symptoms. Interventions comprising an active component, such as videos or quizzes, have proven to be the most effective since they provide an opportunity to practice behavior in a relatively engaging environment and learn by doing. |
| 109 | Arthur et al | 2022 | mental health literary framework |  |
| 110 | Lexen et al. | 2021 | Mental health literacy framework | Research is needed on how to improve employers’ and rehabilitation professionals’ mental health literacy related to the return to work process of persons with MH problems |
| 111 | Amaral et al | 2020 | European Framework for Action on Mental Health and Well-being | Establishes as goals to warrant effective implementation of policies contributing to promoting mental health and to promote mental health by integrating mental health in all policies as goal |
| 112 | Zare et al. | 2021 | Mental health literacy framework | MHL is fundamental to promoting young people’s mental health, early intervention and ongoing care. It is vital to diagnose physical symptoms, facilitate early recognition of mental health problems and promote help-seeking. MHL in youth is essential for the development of protective factors related to social and emotional well-being. Developing an understanding of mental health and mental illness reduces self-stigma |
| 113 | Liu | 2021 | Mental Health Literacy framework | Education offers an effective tool in shaping future nurses’ attitudes toward people with mental disorders and promoting mental health nursing. Although there is a recognized need to enhance MHL among nursing students, the current comprehensive nursing curriculum has been criticized for its minimal representation of mental health content. |
| 114 | Liddle et al. | 2021 | Mental health literacy in sports context | In Australia, around 70% of adolescents participate in organized sport each year (Australian Bureau of Statistics, 2015), with individuals between 12 and 17 years of age participating in more than 6 hr of organized sport on average per week. This presents an ideal environment and opportunity for the promotion, prevention, and intervention of mental health among male adolescents. |
| 115 | Gratwick-Sarll and Bentley | 2014 | Mental health literacy framework | The ability to recognize bulimic-type eating disorders has been identified as a significant factor for improving early intervention and treatment seeking. Currently, public awareness, as well as self-recognition of disordered eating, has been found to be quite poor among individuals experiencing bulimic-type eating |
| 116 | Ayano et al. | 2017 | Mental health literacy framework | mental disorders in low- and middle income countries (LMICs) |
| 117 | Bonnie et al. | 2019 | Mental health literacy framework | Online education intervention for mental health |
| 118 | Slewa-Younan | 202 | Mental health literacy framework | MHL of culturally diverse communities is an emerging area of research. Specific to this study is the evidence related to Arabic speaking refugee groups, which has demonstrated that differing levels of knowledge and beliefs about the nature and the management of mental health problems may act as barriers to help-seeking. It is postulated that culturally tailored mental health education and promotion programs addressing these barriers are required |
| 119 | Reavley et al. | 2014 | Mental health literacy framework | MindWise, a multifaceted intervention, could improve mental health literacy, facilitate help seeking and reduce psychological distress and alcohol misuse in staff and students of Victoria University (VU) in metropolitan Melbourne, Australia |
| 120 | Fung et al. | 2016 | Mental health literacy framework | barrier to treatment of mental disorders is public stigma — the reaction of the general public to people with mental illness |
| 121 | Bella-Awusah | 2014 | Mental health literacy framework | Educational programmes to improve mental health illiteracy and reduce stigma |
| 122 | Edgar et al. | 2021 | Mental Health First Aid (MHFA) | MHFA training for physiotherapy students’ attitudes toward psychiatry and mental health |
| 123 | Mohatt et al | 2017 | Mental Health First Aid (MHFA) | Existing training programs for military personnel such as Battlemind16 and the Comprehensive Soldier Fitness Program17 were designed to help soldiers build their resilience and reintegrate into their communities, Military MHFA (M-MHFA) is designed to increase the system of laypeople (both military and civilian) ready to provide basic support and assistance to others with developing mental health problems |
| 124 | Farooq et al. | 2021 | Mental health literacy framework | MHL programs need to promote awareness about and acceptance of persons with mental illness. They should commence in schools and continue into colleges and universities. In Pakistan, neither the school nor college curricula pay adequate attention to mental health. Therefore, these younger populations lack knowledge and optimistic attitudes towards treatment and counselling services |
| 125 | o'Reilly et al. | 2011 | Mental Health First Aid (MHFA) | A training programme in how to support someone in a mental health crisis or who is developing a mental disorder; Pharmacists are frontline primary healthcare workers and it is essential that they are adequately trained in mental health to ensure they are confident and have the skills to effectively communicate with consumers with a mental illness. |
| 126 | Gurung et al | 2020 | Mental Health First Aid (MHFA) training | Effectiveness of bilingual (English/Nepali) MHFA training offered with and without a culturally-appropriate orientation to mental health terminology and concepts used in conventional approaches to mental health care in the United States |
| 127 | Jorm et al | 2010 | Mental health first aid | E-learning has been applied to both medical education and to psychological treatments and can produce effects comparable to instructor-delivered training or face-to-face therapy |
| 128 | Lam et al. | 2010 | Mental health first aid | Mental health literacy in Chinese community in Australia, a suitable training program is required for improving the necessary confidence and skills to provide basic help. |
| 129 | Jordans et al | 2012 | Mental health policy | in Nepal there was no coordinated psychosocial and mental health strategy in disaster response to date in Nepal; to evaluate the impact of brief two-day training courses on the knowledge and attitudes of front-line level staff on the integration of mental health and psychosocial support in disaster relief and humanitarian efforts |
| 130 | Guajardo et al | 2019 | Mental health literacy | Adolescents showing symptoms of mental illness are supported to engage appropriate help‐seeking and access effective treatment interventions early in the course of illness. Research on understanding help-seeking in young people identify three possible factors that may play a role: mental health literacy, stigma and social support |
| 131 | Patafio et al | 2021 | Mental health literacy framework | Poor mental health literacy exacerbates this culture of mental toughness and its subsequent impact on help-seeking within the sporting context, wherein a lack of knowledge to guide and promote positive attitudes toward mental health and help-seeking within sporting clubs restricts player’s ability to seek advice and healthily address problems. |
| 132 | Tuijunam et al | 2022 | Mental health literacy and stigma | game-based school program, Moving Stories, which combines mental health literacy training for depression with contact with someone with lived experience, both in the digital and nondigital world |
| 133 | Tay et al. | 2019 | NIL | NIL |
| 134 | Zamorski et al. | 2012 | Third-location decompression (TLD) programs 6 and psychoeducational programs | The U.S. Army’s Battlemind training, which has been shown to improve postdeployment well-being.7 The Battlemind program is a cognitive and skill-based program that reframes transition difficulties as a failure to adapt skills learned in combat to the home environment. |
| 135 | oduguwa et al | 2017 | Protest strategy; anti-stigma strategies--education strategy; Contact strategy | Protest strategy is often described as a responsive approach that aims to challenge misrepresentations and negative beliefs about mental illness projected by the media and accepted by the public, but not necessarily replacing these unfavourable expressions with positive and factual information about mental illness. Research has shown that anti-stigma strategies using protest have been effective but may have potential rebound effects. Education strategy aims to provide factual information about mental illness and has been shown to improve the attitude of its target audience towards persons with mental illness, howbeit; the effects may not be sustained for a long period of time. Contact strategy provides a platform for the public to meet and interact with persons with mental illnesses who are doing well on their jobs and are able to interact well with their neighbours. |
| 136 | Pinto-Foltz et al | 2011 | Developmental theories, Pescosolido, Martin, Lang, and Olafsdottir’s (2008) Framework Integrating Normative Influences on Stigma (FINIS) | Erikson’s stages of psychosocial development and Bronfrenbrenner’s bioecological model, underscore the contributions of peers and influential adults on adolescent help seeking behavior. Within the context of mental illness stigma, Pescosolido, Martin, Lang, and Olafsdottir’s (2008) Framework Integrating Normative Influences on Stigma (FINIS) illustrates the multiple levels, beyond the individual, that influence mental illness stigma |
| 137 | Bjørnsen et al | 2018 | Mental health literacy framework | The first component of MHL in Kutcher et al.’s definition is referred to in this study as positive MHL (1), understanding how to obtain and maintain good mental health. This component (1) is essential from a health promotion perspective in which the focus is on knowledge of good mental health rather than on mental disorders |
| 138 | Wilcox et al | 2023 | teen Mental Health First Aid (tMHFA) | First school-based program to show effective stigma reduction |
| 139 | Crooks et al | 2018 | Mental Health First Aid First Nations (MHFAFN) | Undertake a feasibility study of the MHFAFN to assess the acceptability of the intervention and cultural adaptation, and preliminary participant outcomes. |
| 140 | Loureiro | 2020 | Mental Health First Aid | considers health literacy, including mental health literacy (MHL), as one of the most important social health determinants, directly impacting the health and well-being of populations Program |
| 141 | Payne et al | 2001 | National Service Framework for Mental Health | NHS Direct is specifically mentioned in the National Service Framework for Mental Health as improving 24-hour access to mental health services. This states that any individual should be able to use NHS Direct, as it develops, for first-level advice and referral on to specialist helplines or local service. It is also ideally placed to address other key standards in the Framework, in particular mental health promotion, support for people who care for those with mental health problems, and the reduction in the suicide rate. |
| 142 | Gargia-Ortega et al | 2013 | Canadian Collaborative Mental Health Initiative | Canadian initiatives to enhance primary mental health care capacity include the Canadian Collaborative Mental Health Initiative nationally and provincially, and the Practice Support Program (PSP) in British Columbia, among others. |
| 143 | DeSocio et al. | 2006 | Mental health literacy framework | mental health education important for school-age children |
| 144 | Repp et al. | 2019 | Mental Health First Aid  (MHFA) | MHFA evaluations have been limited by their lack of racial-ethnic diversity |

Multimedia Appendix 2.

Table S3. Effects of traditional, face-to-face and DMHL interventions on mental health

| **Mental health literacy interventions** | **Mental Health** | | | | | |
| --- | --- | --- | --- | --- | --- | --- |
|  | *K^a^* | N^b^ | smd^c^ | 95% *C. I.^d^* | *Q^e^* | *I^2f^* |
| Traditional, face-to-face | 48 | 27,692 | 0.33^**^ | [0.15, 0.46] | 1713.21^*^ | 97.22 |
| DMHL | 79 | 13,721 | 0.42^**^ | [0.17, 0.71] | 1753.11^*^ | 96.91 |

Table S4. Effects of DMHL interventions on uptake

| **DMHL interventions** | **Uptake** | | | | | |
| --- | --- | --- | --- | --- | --- | --- |
|  | *K^a^* | N^b^ | Odd-ratio^c^ | 95% *C. I.^d^* | *Q^e^* | *I^2f^* |
| Intervention vs. control/other treatment conditions | 87 | 21,213 | 0.998^**^ | [0.91, 1.03] | 297.45^*^ | 94.00 |

^a^number of effect sizes.

^b^sample size.

^c^effect sizes.

^d^confidence interval.

^e^ratio of observed variance to within-study variance.

^f^I^2^ = percentage of observed variation that can be attributed to the actual differences between studies, rather than within-study variance.

^*^*P*< .0001

With high heterogeneity observed among traditional, face-to-face mental health literacy and DMHL interventions, we used a random-effects model in conducting meta-analyses and meta-regressions on moderating effects of the effectiveness of DMHL interventions on mental health. The random-effect model has the assumption of study heterogeneity—that is, the amount of variation in effect sizes that is derived from both study error and true study heterogeneity. The latter stems from variation in study populations, study procedures, measures, and settings. To ascertain true heterogeneity in effect sizes, we used the Q-statistic, which calculates the ratio of observed variation to within-study variance. A significant Q-statistic provides evidence that the included studies do not share a common effect size. A caveat about Q-statistic is that it underestimates heterogeneity in small samples and overestimates that in large samples. Thus, we included the I^2^ statistic, which is a percentage that indicates the proportion of observed variation that is attributed to the actual differences between studies, rather than within-study variance. I^2^ of 25, 50, and 75 percent represent low, moderate, and high variance, respectively. Compared to the Q-statistic, I^2^ is not sensitive to the number of studies included and allows for CIs to be calculated [90].
