# Supplementary material for: The Effect of Digital Mental Health Literacy Interventions on Mental Health: Systematic Review and Meta-Analysis
Source: J Med Internet Res. 2024 Feb 29;26:e51268. doi: 10.2196/51268 (PMC10941000; doi:10.2196/51268)
Supplement: Multimedia Appendix 3 [file jmir_v26i1e51268_app3.docx]

Multimedia Appendix 3.

Table S6. Summary and sample statistics of DMHL interventions

|  | Control | | | Experimental | | | Alternative Treatment | | |  |  |
| --- | --- | --- | --- | --- | --- | --- | --- | --- | --- | --- | --- |
| **Study** | **M^a^** | **SD^b^** | **Total^c^** | **M^a^** | **SD^b^** | **Total^c^** | **M^a^** | **SD^b^** | **Total^c^** | **Weight** | **Standardized Mean Difference^d^** |
| Ali et al. 2014 | - | - | - | 36.93 | 10.4 | 120 | 37.5 | 11.2 | 121 | 1.94 | 0.117^3^ |
| Alvarez-Jimenez et al. 2021 |  |  |  | 17.90 | 0.568 | 86 | 17.80 | 0.59 | 84 | 1.910 | 0.518^3^ |
| Andrews et al. 2022 | 4.36 | 4.71 | 1267 | 4.78 | 5.14 | 1272 | - | - | - | 2.030 | 0.037^2^ |
| Arjadi et al. 2018 | - | - | - | 18·01 | 5·05 | 154 | 17·92 | 5·38 | 159 | 1.97 | 0.240^3^ |
| Bakker et al. 2018 | 56.44 | 15.74 | 63 | 59.73 | 14.23 | 54 | - | - | - | 1.940 | 0.245^2^ |
| Baumeister et al. 2021 | 9.93 | 5.3 | 105 | 8.11 | 4.49 | 104 | - | - | - | 1.930 | 0.364^2^ |
| Blaney et al. 2021 | - | - | - | (59.73)^e^  54.93 | (7.44)^e^  7.87 | (28)^e^  28 | - | - | - | 1.380 | 0.713^1^ |
| Carl et al. 2020 | 16.70 | 6.14 | 82 | 17.43 | 6.33 | 81 | - | - | - | 1.950 | 0.574^2^ |
| Cernvall et al. 2018 | - | - | - | (14.12)^e^  12.09 | (6.23)^e^  6.83 | (11)^e^  11 | - | - | - | 0.810 | 0.282^1^ |
| Cieslak et al. 2016 | - | - | - | 2.37 | 0.64 | 81 | 2.21 | 0.54 | 87 | 1.90 | 0.280^3^ |
| De Silva 2022 | 9.40 | 0.39 | 79 | 9.56 | 0.21 | 97 | - | - | - | 1.91 | 0.906^2^ |
| Ebert et al. 2021 |  |  |  |  |  |  |  |  |  | 1.950 | 0.570^2^ |
| Ebert et al. 2016 | 15 | 5.86 | 132 | 11.7 | 5.50 | 131 | - | - | - | 1.980 | 0.559^2^ |
| Espie et al. 2019 | - | - | - | 21.37 | 9.18 | 858 | 19.16 | 9.08 | 853 | 1.030 | 0.366^3^ |
| Eustis et al. 2018 | 27.64 | 9.15 | 77 | 26.73 | 8.09 | 78 | - | - | - | 1.890 | 0.349^2^ |
| Fogarty et al. 2017 | - | - | - | (21.13)^e^  17.38 | (0.284)^e^  0.545 | (144)^e^  144 | - | - | - | 1.880 | 0.510^1^ |
| Goetz et al. 2020 | - | - | - | (28.82)^e^  22.89 | (7.40)^e^  7.19 | (39)^e^  39 | - | - | - | 1.720 | 0.122^1^ |
| Graham et al. 2020 | 11.3 | 5.42 | 58 | 8.7 | 4.8 | 64 | - | - | - | 1.880 | 0.628^2^ |
| Harrer et al. 2021 | 30.26 | 9.53 | 100 | 30.85 | 9.21 | 100 | - | - | - | 1.930 | 0.375^2^ |
| Hui et al. 2015 | 2.73 | 1.58 | 62 | 3.4 | 1.1 | 54 | - | - | - | 1.920 | 0.492^2^ |
| Immura et al. 2016 | 11.8 | 6.87 | 559 | 10.67 | 7.22 | 531 | - | - | - | 2.02 | 0.180^2^ |
| Johansson et al. 2022 | 22.55 | 6.25 | 72 | 22.5 | 6.75 | 72 | - | - | - | 1.880 | 0.054^2^ |
| Jones et al. 2020 | - | - | - | (29.94)^e^  30.39 | (10.75)^e^  10.84 | (35)^e^  35 | - | - | - | 1.670 | 0.203^1^ |
| Kawadler et al. 2020 | - | - | - | (47.96)^e^  43.97 | (10.05)^e^  8.8 | (55)^e^  55 | - | - | - | 1.660 | 0.810^1^ |
| Koike et al. 2018 | 12.9 | 3.3 | 87 | 13.8 | 2.85 | 89 | - | - | - | 1.950 | 0.291^2^ |
| Kuhn et al. 2017 | 30.37 | 10.08 | 58 | 30.48 | 10.14 | 62 | - | - | - | 1.850 | 0.410^2^ |
| Lattie et al. 2020 | - | - | - | (11.29)^e^ 12.15 | (4.78)^e^  5.95 | (20)^e^  20 | - | - | - | 1.200 | 0.484^1^ |
| Levin et al. 2016 | - | - | - | 23.34 | 11.12 | 77 | 22.69 | 9.86 | 102 | 1.90 | 0.108^3^ |
| Lintvedt et al. 2013 | 26.54 | 7.35 | 80 | 27.65 | 17.15 | 81 | - | - | - | 1.900 | 0.535^2^ |
| MacLean et al. 2020 | 12.4 | 6.4 | 48 | 11.3 | 6.4 | 47 | - | - | - | 1.810 | 1.820^2^ |
| Mailey et al. 2019 | 15.28 | 6.46 | 72 | 13.75 | 5.85 | 79 | - | - | - | 1.940 | 0.199^2^ |
| Mak et al. 2018 | - | - | - | 2.72 | 0.90 | 753 | 2.69 | 0.89 | 1408 | 2.030 | 0.042^3^ |
| Mehrotra et al. 2018 | - | - | - | (28.32)^e^  26.20 | (7.08)^e^  7.02 | (50)^e^  50 | - | - | - | 1.760 | 0.793^1^ |
| Milgrom et al. 2020 | 36.69 | 10.75 | 20 | 34.75 | 12.21 | 21 | - | - | - | 1.570 | 0.432^2^ |
| Moeini et al. 2019 | 6.58 | 3.64 | 50 | 6.19 | 3.48 | 45 | - | - | - | 1.860 | 0.412^2^ |
| Moessner et al. 2016 | 2.28 | 2.71 | 593 | 3.82 | 3.36 | 238 | - | - | - | 1.990 | 0.480^2^ |
| Moser et al. 2019 | - | - | - | 15.22 | 4.39 | 16 | 15.61 | 4.13 | 35 | 1.810 | 0.292^3^ |
| Nguyen-Feng et al. 2017 | 1.90 | 0.58 | 243 | 1.83 | 0.57 | 122 | - | - | - | 1.980 | 0.134^2^ |
| O'Dea et al. 2020 | 16.70 | 6.14 | 82 | 17.43 | 5.96 | 80 | - | - | - | 1.920 | 0.125^1^ |
| O'Dea et al. 2021 | 11.63 | 23.66 | 936 | 10.16 | 18.47 | 444 | - | - | - | 2.030 | 0.094^2^ |
| Persson Asplund et al.2018 | - | - | - | 11.35 | 4.20 | 59 | 14.47 | 4.87 | 58 | 1.850 | 0.722^3^ |
| Radovic et al. 2021 | - | - | - | 1.2 | 3.55 | 5 | -0.5 | 6.95 | 17 | 1.520 | 0.358^3^ |
| Rodante et al. 2020 | 0.721 | 1.66 | 9 | 0.642 | 1.14 | 9 | - | - | - | 1.130 | 0.558^2^ |
| Shehadeh et al. 2019 | - | - | - | (13.41)^e^  8.65 | (4.75)^e^  5.82 | (26)^e^  26 | - | - | - | 1.860 | 0.394^1^ |
| Sin et al. 2022 | 62.97 | 18.70 | 159 | 64.53 | 18.03 | 161 | - | - | - | 1.980 | 0.089^2^ |
| Tighe et al. 2017 | 28.06 | 5.68 | 30 | 28.21 | 5.81 | 31 | - | - | - | 1.890 | 0.680^2^ |
| Tighe et al. 2017 | - | - | - | (29.28)^e^  26.34 | (5.83)^e^  6.08 | (61)^e^  61 | - | - | - | 1.690 | 0.265^1^ |
| Van Voorhees et al. 2012 | - | - | - | (29.5)^e^  28.43 | (8.53)^e^  8.97 | (50)^e^  41 | - | - | - | 1.630 | 0.330^1^ |
| Vechiu 2021 | 3.67 | 5.82 | 57 | 23.44 | 5.14 | 53 | - | - | - | 1.840 | 0.484^2^ |
| Yamaguchi et al. 2019 | 9.92 | 3.36 | 65 | 8.38 | 3.43 | 56 | - | - | - | 1.950 | 0.308^2^ |
| Yamaguchi et al. 2019 | - | - | - | 8.38 | 3.43 | 56 | 7.99 | 3.14 | 66 | 1.950 | 0.087^3^ |
| Zwerenz et al.2019 | - | - | - | 31.90 | 9.47 | 108 | 34.0 | 9.67 | 107 | 1.940 | 0.353^3^ |
|  |  |  |  |  |  |  |  |  |  |  |  |
| Brooks et al.2023 | - | - | - | (35.1)^e^  34.9 | (9.6)^e^  8.7 | (78)^e^  78 | - | - | - | 1.031 | 0.022^1^ |
| Tay et al.2022 | 87.69 | 16.13 | 89 | ( 88.4)^e^  87.27 | (13.78)^e^  15.25 | (90)^e^  89 | - | - | - | 0.386 | 0.027^2^ |

*^a^*mean (M)

^b^standard deviation (SD)

^c^Total number of participants according to their group (control, experimental, alternative treatment)

^d^Standardized Mean Differences (SMD) are provided, where study design/comparison of conditions

^e^Pre-score

^1^SMD between pre and post DMHL intervention

^2^SMD between DMHL vs. waitlist control

^3^SMD between DMHL vs. Non-DMHL (all DMHL interventions were DMHL PLUS, and there are no studies comparing DMHL ONLY and non-DMHL).

First, different effect sizes were used to conduct analyses with mental health separately for cases examining more than one study designs: (a) pre-post DMHL interventions, (b) (wait-list) control vs. DMHL interventions, and (c) DMHL intervention vs. non-DMHL interventions; for cases that included more than one DMHL component: (a) DMHL ONLY vs. waitlist control, (b) DMHL PLUS vs. waitlist control, (c) DMHL ONLY vs. DMHL PLUS, (d) DMHL ONLY vs. non-DMHL, and (e) DMHL PLUS vs. non-DMHL; and for cases examining DMHL interventions administered through multiple platforms: (a) new platforms (i.e., mobile apps, web-based/internet platforms, and social media) and (b) conventional platforms (i.e., films, videos, multimedia, and emails). Second, some studies measured DMHL in a variety of ways, for instance, different combination of DMHL facets were assessed and multiple indicators of mental health were included, providing several effect sizes from the same study. For such cases, we computed the average effect size across all DMHL facets and all measures of the same mental health outcome within a study. Hence, each study contributed only one effect size for the analyses involving DMHL interventions with mental health. Similarly, for studies that assessed multiple indicators of proximal literacy outcomes and distal mental health outcomes, we computed the average effect size across all indicators. Third, a few studies reported multiple effect sizes because they investigated different countries, especially concerning representatives of Western and Eastern cultural contexts. Because cultural contexts were examined as a moderator in our meta-analyses, a single effect size estimate that aggregated the multiple correlation coefficients was not favored and we reported effect size estimates separately [82]. For such cases, more than one set of data was collected from the same study, forcing consideration of issues of statistical dependency that stem from the multiple dependent effect sizes [82]. We used the robust variance estimation to account for non-independent effect sizes, which can also be adjusted to deal with smaller meta-analyses (n < 40; [83]). Pooled effect size of odd-ratios (OR) were used in examining implementation effectiveness of interventions on uptake, and standardized mean differences (SMDs) were used in assessing intervention effectiveness on mental health.
