# Supplementary material for: The Effect of Digital Mental Health Literacy Interventions on Mental Health: Systematic Review and Meta-Analysis
Source: J Med Internet Res. 2024 Feb 29;26:e51268. doi: 10.2196/51268 (PMC10941000; doi:10.2196/51268)
Supplement: Multimedia Appendix 5 [file jmir_v26i1e51268_app5.docx]

Multimedia Appendix 4.

Figure S7. Forest Plot of data investigating DMHL and traditional, face-to-face mental health literacy interventions with mental health

Author(s), Year Standardized Mean Difference [95% CI]


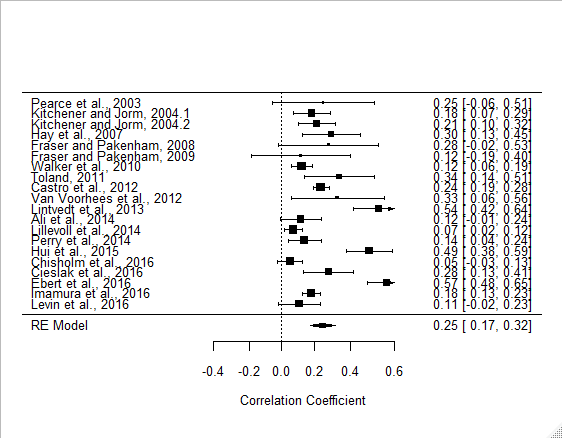


Standardized Mean Difference

Author(s), Year Standardized Mean Difference [95% CI]


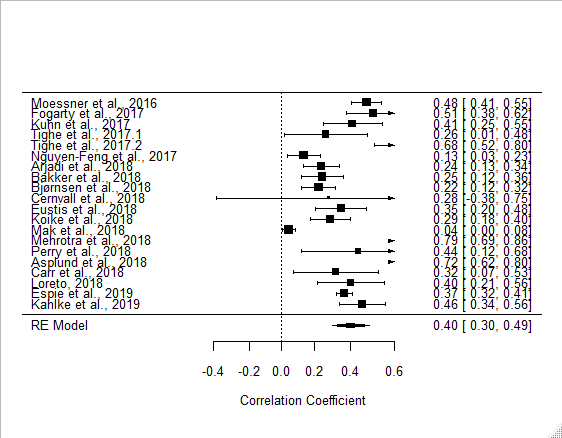


Standardized Mean Difference

[Continue from above]

Author(s), Year Standardized Mean Difference [95% CI]


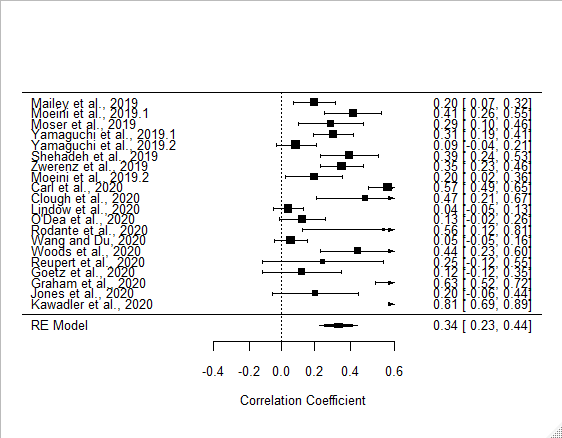


Standardized Mean Difference

Author(s), Year Standardized Mean Difference [95% CI]


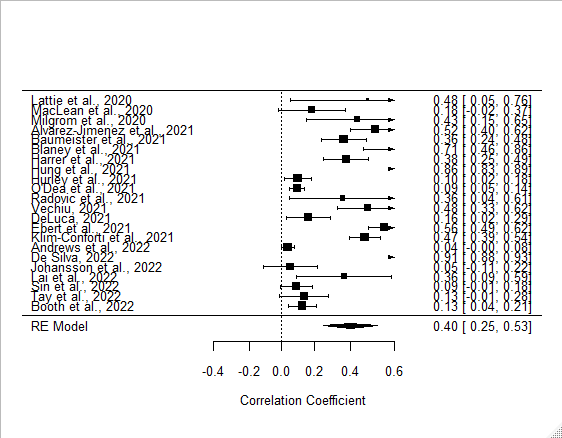


Standardized Mean Difference

[Continue from above]

Author(s), Year Standardized Mean Difference [95% CI]


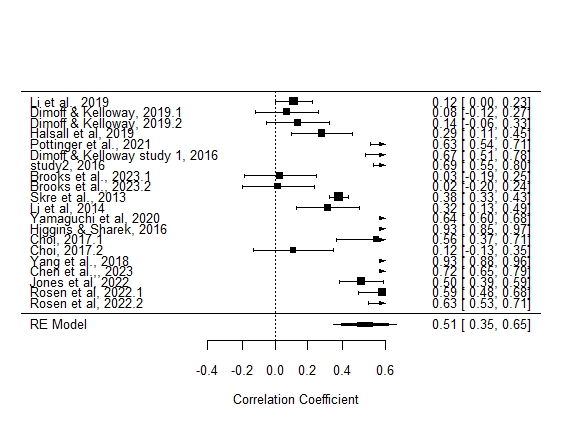


Standardized Mean Difference

Author(s), Year Standardized Mean Difference [95% CI]


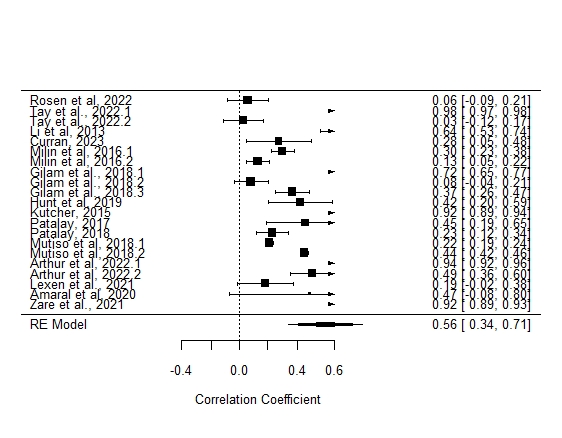


Standardized Mean Difference

[Continue from above]

Author(s), Year Standardized Mean Difference [95% CI]


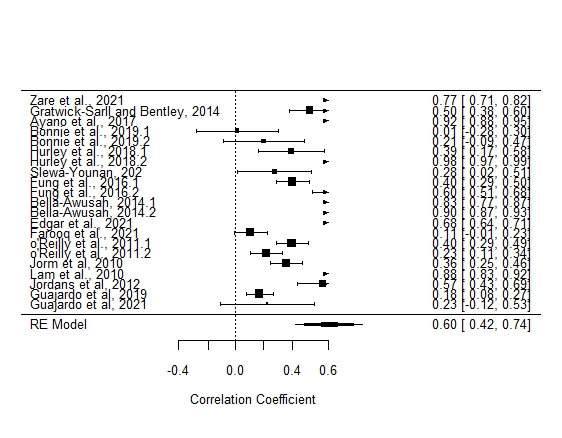


Standardized Mean Difference

Author(s), Year Standardized Mean Difference [95% CI]


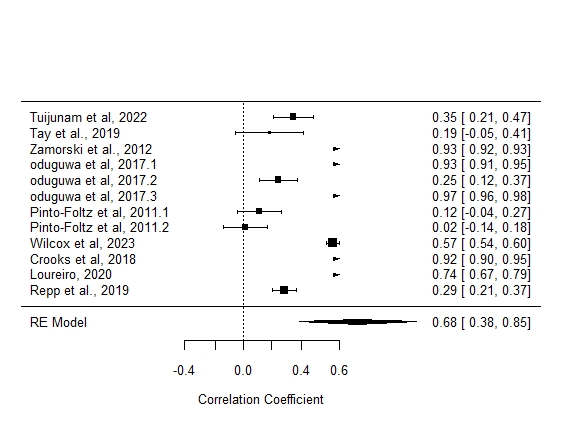


Standardized Mean Difference

In the plot, each study is represented by a point estimate and is bounded by a CI for the effect, and at the bottom of the plot, the summary effect size is represented by the polygon, with its width representing 95% CI. Studies with larger squares contributed more to the summary effect size as compared to other studies. Studies that contributed multiple dependent effect sizes in our meta-analyses were dealt with in three ways following the guidelines provided by Quintana. First, different effect sizes were used to conduct analyses with mental health separately for cases examining more than one study designs: (a) pre-post DMHL interventions, (b) (wait-list) control vs. DMHL interventions, and (c) DMHL intervention vs. non-DMHL interventions; for cases that included more than one DMHL component: (a) DMHL ONLY vs. waitlist control, (b) DMHL PLUS vs. waitlist control, (c) DMHL ONLY vs. DMHL PLUS, (d) DMHL ONLY vs. non-DMHL, and (e) DMHL PLUS vs. non-DMHL; and for cases examining DMHL interventions administered through multiple platforms: (a) new platforms (i.e., mobile apps, web-based/internet platforms, and social media) and (b) conventional platforms (i.e., films, videos, multimedia, and emails).

Second, some studies measured DMHL in a variety of ways, for instance, different combination of DMHL facets were assessed and multiple indicators of mental health were included, providing several effect sizes from the same study. For such cases, we computed the average effect size across all DMHL facets and all measures of the same mental health outcome within a study. Hence, each study contributed only one effect size for the analyses involving DMHL interventions with mental health. Similarly, for studies that assessed multiple indicators of proximal literacy outcomes and distal mental health outcomes, we computed the average effect size across all indicators.

Third, a few studies reported multiple effect sizes because they investigated different countries, especially concerning representatives of Western and Eastern cultural contexts. Because cultural contexts were examined as a moderator in our meta-analyses, a single effect size estimate that aggregated the multiple correlation coefficients was not favored and we reported effect size estimates separately [82]. For such cases, more than one set of data was collected from the same study, forcing consideration of issues of statistical dependency that stem from the multiple dependent effect sizes [82]. We used the robust variance estimation to account for non-independent effect sizes, which can also be adjusted to deal with smaller meta-analyses (n < 40; [83]). Pooled effect size of odd-ratios (OR) were used in examining implementation effectiveness of interventions on uptake, and standardized mean differences (SMDs) were used in assessing intervention effectiveness on mental health.
