# Supplementary material for: The Effect of Digital Mental Health Literacy Interventions on Mental Health: Systematic Review and Meta-Analysis
Source: J Med Internet Res. 2024 Feb 29;26:e51268. doi: 10.2196/51268 (PMC10941000; doi:10.2196/51268)
Supplement: Multimedia Appendix 6 [file jmir_v26i1e51268_app6.docx]

| Table S1. Moderators of DMHL interventions and mental health | | | | | |
| --- | --- | --- | --- | --- | --- |
|  | **Mental** **Health** | | | | |
| **Moderator** | *k* | smd | 95% *C.I.* | Between Group *Q* | *I^2^* |
|  |  |  |  |  |  |
| **Mode** |  |  |  |  |  |
| Traditional, face to face mental health literacy interventions | 102 | 0.33 | [0.15, 0.46] | 4.12 | 97.22 |
| DMHL interventions | 63 | 0.42 | [0.17, 0.71] |  |  |
|  |  |  |  |  |  |
| **DMHL components** |  |  |  |  |  |
| DMHL ONLY vs. waitlist control | 102 | 0.59 | [0.49, 0.91] | 10.05^*^ | 96.21 |
| DMHL PLUS vs. waitlist control |  | 0.45 | [0.001,0.90] |  |  |
| DMHL ONLY vs. DMHL PLUS |  | -0.35 | [-0.60,-0.11 ] |  |  |
| DMHL PLUS vs. non-DMHL |  | -0.33 | [-0.59,-0.07] |  |  |
|  |  |  |  |  |  |
| **Study design** |  |  |  |  |  |
| Pre-post DMHL interventions | 94 | 0.52 | [-0.05,0.79] | 12.09^**^ | 97.22 |
| DMHL vs. waitlist control |  | 0.46 | [-0.13, 0.69] |  |  |
| DMHL vs. non-DMHL interventions |  | -0.33 | [-0.59,-0.07] |  |  |
|  |  |  |  |  |  |
| **Assessment** |  |  |  |  |  |
| Post-intervention | 107 | 0.47 | [0.31, 0.61] | 3.81 | 94.02 |
| Follow-up |  | 0.37 | [0.08, 0.60] |  |  |
|  |  |  |  |  |  |
| **Length of follow-up assessment** | 50 | -0.004 | [-0.01, 0.002] | 1.65 | 98.75 |
|  |  |  |  |  |  |
| **Platform** |  |  |  |  |  |
| New with greater interactivity | 102 | 0.40 | [0.20,0.75] | 2.51 | 95.95 |
| Conventional with limited interactivity |  | 0.39 | [0.15,0.64] |  |  |
|  |  |  |  |  |  |
| **Dosage** |  |  |  |  |  |
| Below 10 weeks | 102 | 0.42 | [0.21,0.85] | 2.13 | 96.87 |
| 10 weeks |  | 0.31 | [0.09,0.52] |  |  |
| Above 10 weeks |  | 0.37 | [0.12,0.73] |  |  |

|  | **Mental** **Health** | | | | |
| --- | --- | --- | --- | --- | --- |
| **Moderator** | *K^a^* | smd^b^ | 95% *C.I.^c^* | Between Group *Q^d^* | *I^2e^* |
|  |  |  |  |  |  |
| **Gender** | 102 | 0.011 | [-0.010,0.023] | 2.01 | 96.10 |
|  |  |  |  |  |  |
| **Developmental stage** |  |  |  |  |  |
| Adolescence | 102 | 0.18 | [0.04, 0.31] | 12.19^***^ | 96.99 |
| Emerging adulthood |  | 0.43 | [0.13, 0.74] |  |  |
| Older adulthood |  | 0.53 | [0.25,0.79] |  |  |
|  |  |  |  |  |  |
| **Severity of mental health condition** | 43 | 0.11 | [-021,0.46] | 0.29 | 94.51 |
|  |  |  |  |  |  |
| **Cultural contexts** |  |  |  |  |  |
| Western | 102 | 0.45 | [0.25, 0.58] | 1.13 | 96.38 |
| Eastern |  | 0.37 | [0.10,0.60] |  |  |
|  |  |  |  |  |  |
| **Uptake** |  |  |  |  |  |
| Proximal MHL outcomes | 31 | -0.002 | [-0.006,0.002] | 1.07 | 96.3 |
| Distal mental health outcomes | 79 | -0.002 | [-0.009,0.006] | 0.17 | 96.5 |
|  |  |  |  |  |  |
| **Samples and proximal MHL outcomes** |  |  |  |  |  |
| DHML interventions with baseline and completer samples that were similar on baseline and demographic measures | 31 | 0.40 | [0.29, 0.51] | 1.19 | 96.26 |
| DMHL interventions that did not provide information on baseline and completer samples’ baseline and demographic measures |  | 0.43 | [0.38, 0.49] |  |  |
| DMHL interventions with baseline and completer samples that were different on baseline and demographic measures |  | 0.27 | [0.07, 0.45] |  |  |
|  |  |  |  |  |  |
| **Samples and distal mental health outcomes** | 79 | 0.64 | [0.49, 0.79] | 5.21 |  |
| DHML interventions with baseline and completer samples that were similar on baseline and demographic measures |  | 0.39 | [0.31, 0.48] |  | 96.32 |
| DMHL interventions that did not provide information on baseline and completer samples’ baseline and demographic measures |  |  |  |  |  |
| DMHL interventions with baseline and completer samples that were different on baseline and demographic measures |  | 0.79 | [0.41, 1.18] |  |  |

^a^number of effect sizes.

^b^ effect sizes.

^c^confidence interval.

*^d^* ratio of observed variation to within-study variance.

*^e^*percentage of observed variation that can be attributed to the actual differences between studies rather than within-study variance.

^*^*P*=.024

^**^*P*=.035

^***^*P* =.003
